# Optimization of B Cell Responses in Human Immune System Mice Through Organoid Based Screening

**DOI:** 10.1002/advs.202524189

**Published:** 2026-06-26

**Authors:** Haiqiao Sun, He Li, Xu Zhu, Zijian Zhang, Deshan Ren, Shuai Ding, Yan Li

**Affiliations:** ^1^ National Resource Center for Mutant Mice MOE Key Laboratory of Model Animal for Disease Study Model Animal Research Center Department of Oncology Nanjing Drum Tower Hospital The Affiliated Hospital of Medical School Nanjing University Nanjing China; ^2^ Department of Rheumatology and Immunology Nanjing Drum Tower Hospital The Affiliated Hospital of Medical School Nanjing University Nanjing China; ^3^ ChemBioMed Interdisciplinary Research Center Nanjing University Nanjing China; ^4^ Wuxi Xishan NJU Institute of Applied Biotechnology Wuxi China

**Keywords:** antibody development, B cells, cytokines, human immune system mice, vaccines

## Abstract

B cells in the human immune system (HIS) mice exhibit weak responses to external antigens, characterized by insufficient antigen‐specific B cell proliferation, low antibody titers, and a lack of differentiation into effector B cell subsets. We hypothesize that this failure is due to the absence of second signals provided by T cells following BCR stimulation. To address this, we developed an organoid screening system using HIS mouse splenocytes to identify missing signals. A combination of IL‐4, IL‐10, IL‐21 together with CD40L was found to drive potent B cell proliferation and differentiation. Further organoid‐based screening revealed that TNF‐α and CpG synergistically promoted IgG class‐switch, and that temporal separation of expansion and differentiation signals enhanced B cell responses. Translating these findings in vivo, CpG‐adjuvanted vaccination followed by sequential i.v. delivery of expansion and differentiation cytokine mixtures induced antigen‐specific B cell expansion, B cell differentiation, and IgG class‐switch in HIS mice without affecting non‐specific B cells. Sorting RBD‐specific B cells from immunized mice yielded recombinant antibodies with high binding affinity and neutralizing activity against SARS‐CoV‐2 pseudovirus. Our study establishes a spleen organoid platform for screening factors that influence B cell responses in HIS mice and provides a generalizable strategy to obtain fully human antibodies for therapeutic development.

## Graphical abstract

Human immune system (HIS) mice show limited B cell responses to immunization. Using HIS mouse spleen organoids, this study identifies cytokine combinations and innate immune signals that drive human B cell expansion, differentiation, and class‐switch. Applying these signals in vivo through temporal separation enables antigen‐specific IgG responses and supports the isolation of fully human neutralizing antibodies.

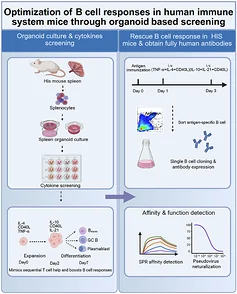

## 1 Introduction

Human immune system (HIS) mice are an important tool in translational medicine and are used to reconstruct the human immune system by transplanting human hematopoietic stem cells into immunodeficient mice, such as NOG (NOD.Cg‐*Prkdc^scid^ Il2rg^tm1Sug^*/JicTac) [1], BRG (C.Cg‐*Rag2^tm1Fwa^ Il2rg^tm1Sug^*/JicTac) [2], NSG (NOD.Cg‐*Prkdc^scid^ Il2rg^tm1Wjl^*/SzJ) [3], or NCG (NOD‐*Prkdc^scid^ Il2rg^tm1/Bcgen^*) [4]. This model provides a unique in vivo platform for studying human‐specific immune responses [5]. However, despite the successful reconstruction of multiple human immune cell populations, including B cells, the functional capacity of B cells in HIS mice falls short of expectations [5, 6]. Specifically, these mice exhibit a lack of antigen‐specific B cell responses, low antibody production levels, and inadequate differentiation of effector B cell subsets [6, 7, 8, 9, 10]. These functional defects severely limit the applicability of HIS mice in key research areas, including vaccine immune evaluations, therapeutic antibody development, and autoimmune disease modeling.

In T‐dependent B cell responses, B cell activation requires two synergistic signals. First, antigen binding to the B cell receptor (BCR) triggers ITAM‐mediated phosphorylation by kinases such as Syk and Btk, activating downstream transcription factors including NF‐κB and NFAT to initiate B cell activation [11, 12]. This signal alone is usually insufficient, and a second signal from activated CD4^+^ helper T cells is required. Through co‐stimulatory and cytokine signals, T cells activate JAK‐STAT and other pathways that drive B cell proliferation, differentiation, and class‐switch recombination [13, 14, 15, 16]. Among membrane‐bound signals, CD40L–CD40 is the predominant co‐stimulatory pathway, activating NF‐κB and MAPK via TRAF proteins and promoting B cell expansion and affinity maturation, while other pairs such as ICOS–ICOSL and OX40–OX40L also contribute to T–B cell cooperation [17, 18].

In HIS mice, abnormalities in CD4^+^ T cell function are thought to be an important factor contributing to defects in B cell responses [5, 6]. Although there is evidence that human T cells in HIS mice can respond to antigen stimulation, they face multiple constraints [19, 20]. First, human T cells undergo selection in a murine thymic environment, which may result in “dual” human–mouse MHC restriction and suboptimal compatibility between T cell receptors (TCRs) and human MHC molecules [21]. This species mismatch can limit efficient recognition of peptide–MHC II complexes on human B cells and compromise cognate T–B interactions [22]. Second, key cytokines such as IL‐7 and IL‐15 are essential for T cell development, survival, and memory formation, but show limited cross‐species reactivity from mouse to human, thereby restricting the activation, expansion, and long‐term maintenance of CD4^+^ T cells [23]. In addition, lymph node development is compromised due to *Il2rg* knockout, and dendritic cells (DC) are predominantly of murine origin, so these xenogeneic antigen‐presenting cells may not provide optimal priming and co‐stimulatory signals for human CD4^+^ T cells [24, 25]. Together, these issues, including thymic selection constraints, suboptimal lymphoid organogenesis, DC defects, and imperfect human–mouse cytokine interactions, make robust antigen‐specific CD4^+^ T cell activation, expansion, and persistence a long‐standing challenge in HIS mice that is comparable to the induction of strong antigen‐specific antibody responses. Advances in HIS mouse engineering have improved human B cell responses through several strategies, including introduction of human cytokines to support B cell and DC development [25, 26, 27, 28, 29], reconstruction of HLA‐mediated T cell selection [8, 21, 22, 30], restoration of lymph node architecture [24], and use of optimized adjuvants [31]. However, these improvements alone remain insufficient to establish robust antigen‐specific B cell immunity.

Based on the mechanisms of B cell activation and the T cell functional defects in HIS mice, we hypothesize that by exogenously supplementing key second signal molecules that CD4^+^ T cells would typically provide, it is possible to bypass the T cell functional defects and directly activate antigen primed B cells. This strategy would target B cells upregulating a series of cytokine receptors after receiving BCR signals to avoid non‐specific activation [12, 32].

To test this hypothesis, we first established an in vitro screening platform using spleen (SP) organoids from HIS mice to evaluate the effects of different cytokines and co‐stimulatory molecules, including IL‐2, IL‐4, IL‐6, IL‐10, IL‐17, IL‐21, and CD40L, on B cell activation and differentiation. Through this platform, we further identified innate immune signals such as TNF‐α and CpG that promote antibody class‐switch, and found that temporal separation of expansion and differentiation signals enhanced B cell responses, reflecting the sequential nature of T cell help during physiological B cell responses. Second, we optimized in vivo delivery by administering selected second‐signal molecules as recombinant proteins rather than through hydrodynamic injection of cytokine‐encoding plasmids, to avoid non‐specific inflammatory responses. Finally, we implemented a phased immunization strategy in which B cells are first engaged by cognate antigens and subsequently exposed to sequentially delivered second‐signal molecules to selectively support their activation, expansion, class‐switch, and differentiation. Using this framework, we demonstrate that exogenous provision of temporally optimized second signals can functionally compensate for CD4^+^ T cell defects in HIS mice, enabling antigen‐specific IgG responses and the generation of fully human monoclonal antibodies with neutralizing activity.

## 2 Results

### 2.1 Establishment of HIS Mouse SP Organoids

**FIGURE 1:**
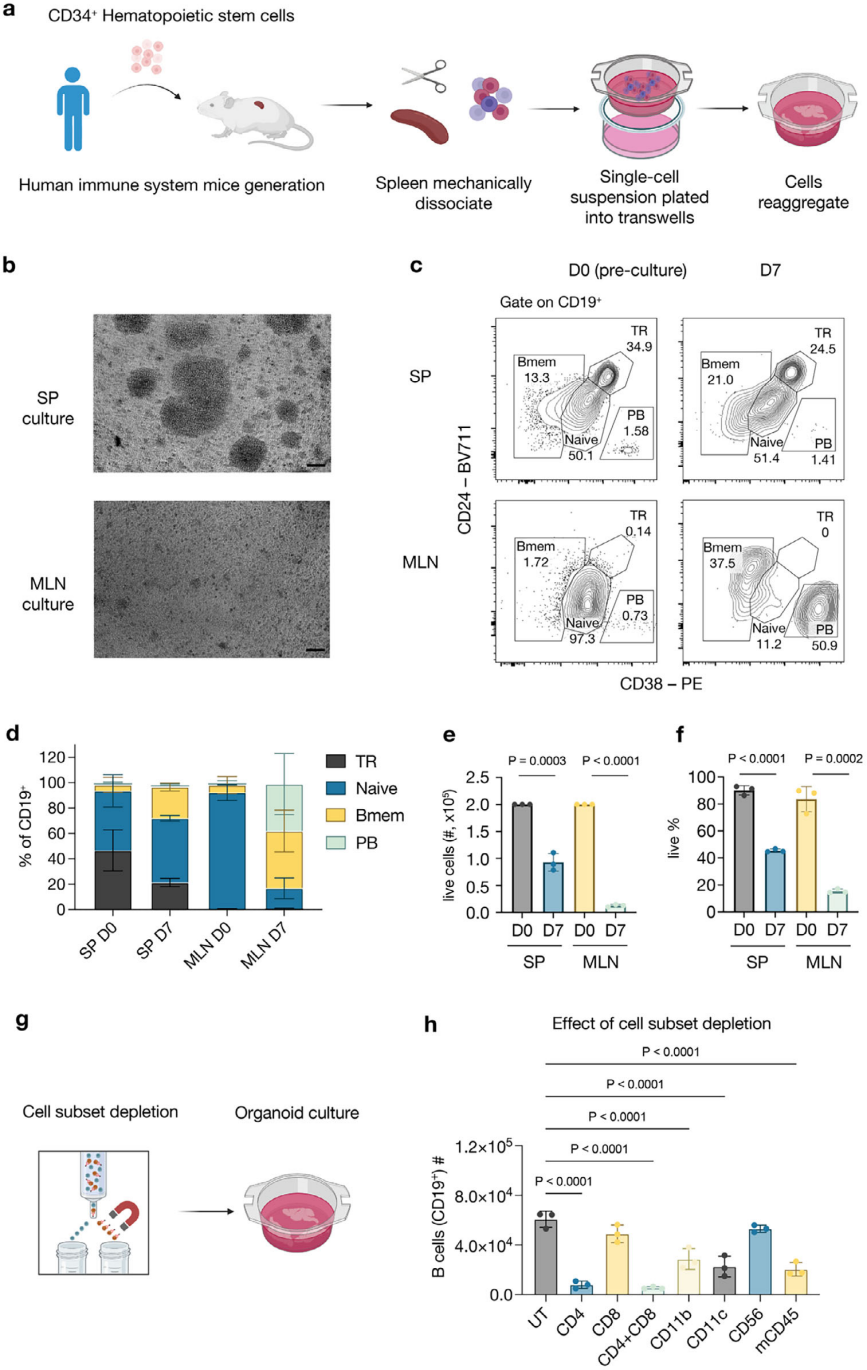
Establishment of HIS mouse SP organoids. (a) Schematic overview of the procedure used to generate SP organoids from HIS mice. HIS mouse SP and mesenteric lymph node (MLN) cells were mechanically dissociated and cultured on permeable membrane plates (Transwells) at a density of 2 × 10^5^ cells per well. (b) Representative images showing the reaggregation of cells into organoid‐like structures after 4 days of culture. Scale bar: 10 µm. (c) Flow cytometry analysis of B cell subsets in SP and MLN‐derived cultures on day 0 (D0) and day 7 (D7) (SP, *n =* 3; MLN, *n =* 6). (d) Quantification of B cell subsets (TR, Naive, Bmem, and PB) in SP and MLN cultures on D0 and D7 (*n =* 3). (e) Total live cell counts on D0 and D7 of SP and MLN‐derived cultures. (f) Live cell percentage on D0 and D7 of SP and MLN‐derived cultures (*n =* 3). (g) Schematic of magnetic bead cell subsets depletion experiment. (h) Quantification of B cell numbers (CD19^+^) in HIS mouse SP organoids after magnetic bead depletion of the indicated immune cell subsets (CD4, CD8, CD4+CD8, CD11b, CD11c, CD56, and mouse CD45(mCD45)) compared to undepleted controls. Organoids were cultured for seven days before analysis. Data are presented as mean ± s.d. Statistical significance was calculated by unpaired t‐test in (e,f) and one‐way ANOVA in (g). *P* < 0.05 was shown.

To screen for cytokine combinations mimicking the second signals required for B cell activation, we sought to establish an organoid system to reduce the cost of HIS mice. Previous studies have demonstrated that human tonsillar lymphocytes can reaggregate and form organoid‐like structures in vitro [33]. Drawing inspiration from this, we isolated SP and mesenteric lymph node (MLN) cells from 10–20‐week‐old HIS mice and cultured them at a density of 2 × 10^5^ cells per well on permeable membrane plates (Transwells) (Figure 1a). After four days of culture, we observed the reaggregation of cells, with SP‐derived clusters being larger in size compared to those from MLN (Figure 1b). After seven days of culture, we performed flow cytometry to analyze the B cell phenotypes in the cultures. In SP‐derived cultures, 24.5% of the B cells had differentiated into memory B (Bmem) cells, while transitional B cells (TR) were reduced. The proportion of Naïve B cells (Naive) remained unchanged, and no significant expansion of plasmablasts (PB) was observed. This indicates that some naïve B cells were activated by exogenous antigens in the culture medium, leading to differentiation into Bmem cells, while overall phenotypic stability was maintained (Figure 1c,d). In contrast, in MLN‐derived cultures, the proportion of Naïve B cells significantly decreased (91.2% vs. 16.2%), and there was a substantial increase in both Bmem (45.1%) and PB (37%), suggesting that Naïve B cells were activated and differentiated into Bmem and PB (Figure 1c,d). In SP cultures, a 45% cell viability was maintained after seven days, with a cell count of approximately 1 × 10^5^, sufficient for downstream flow cytometry analysis (Figure 1e,f). Meanwhile, the SP cultures exhibited superior cell viability compared with the MLN cultures (45.4% vs. 15.4%) (Figure 1e,f). Overall, we found that SP‐derived organoid cultures displayed stable B cell phenotypes and were easily obtainable. As such, we selected SP as the preferred cell source for establishing the in vitro screening system.

To investigate whether cellular components within HIS mouse SP organoids contribute to B cell survival, we used magnetic bead depletion to remove different immune cell populations from HIS mouse splenocytes, including CD4^+^ T cells, CD8^+^ T cells, CD4^+^/CD8^+^ T cells, CD11b^+^ cells, CD11c^+^ cells, CD56^+^ natural killer (NK) cells, and mouse CD45^+^ cells. After seven days of culture, we found that depletion of almost all immune cell subsets led to reduced B cell survival, with the exception of CD8^+^ T cells and NK cells. Among these, removal of CD4^+^ T cells resulted in the most pronounced decrease in B cell numbers (Figure 1g). These results indicate that diverse immune cell subsets within the organoids support B cell survival in HIS mouse SP organoids.

### 2.2 Identification of Potential Second Signal Components for Human B Cell Activation using HIS Mouse SP Organoids

**FIGURE 2:**
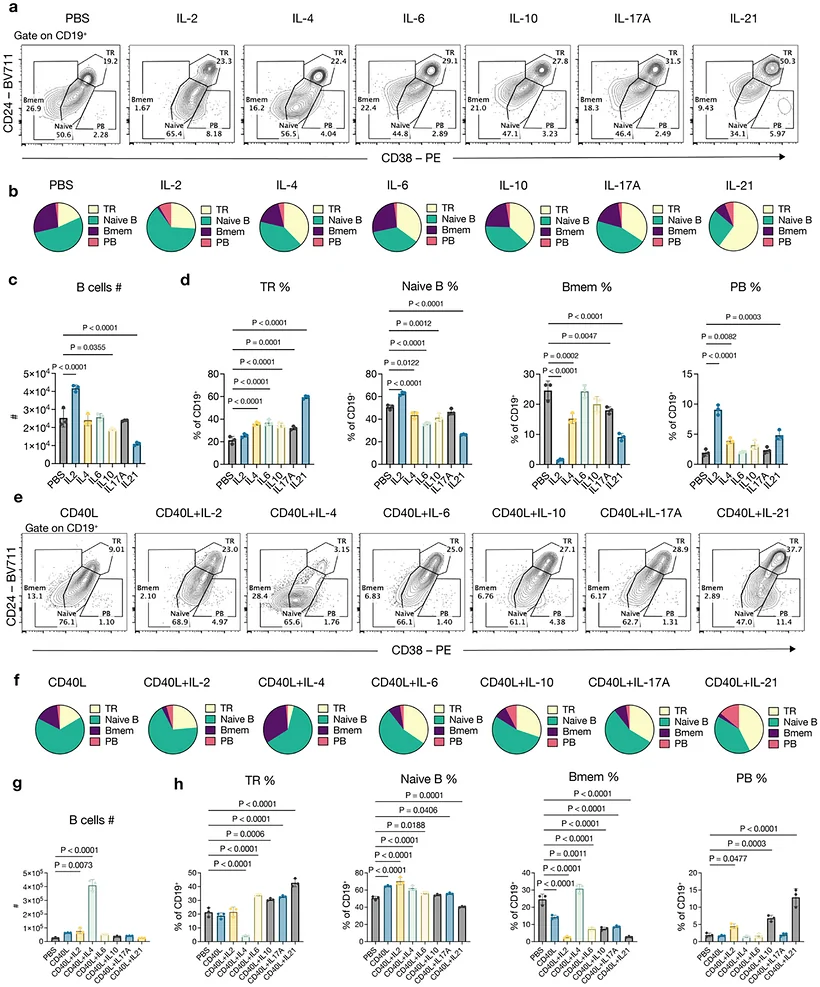
Identification of potential cytokine‐induced second signal components for human B cell activation using HIS mouse SP organoids. (a) Flow cytometry analysis of B cell subsets (Naive, TR, Bmem, and PB) in HIS mouse SP organoids treated with different cytokines (IL‐2, IL‐4, IL‐6, IL‐10, IL‐17, and IL‐21) compared to PBS controls. The proportions of B cell subsets were measured based on CD24 and CD38 expression. (b) Pie charts illustrating the distribution of B cell subsets (Naive, TR, Bmem, and PB) in SP organoids treated with the indicated cytokines (*n =* 3). (c) Quantification of B cell numbers (CD19^+^) in HIS mouse SP organoids after cytokine treatment. d) Quantification of B cell subsets (TR, Naive, Bmem, and PB) as a percentage of CD19^+^ cells in SP organoids treated with individual cytokines. (e) Flow cytometry analysis of B cell subsets in SP organoids treated with CD40L and cytokine combinations (CD40L + IL‐2, CD40L + IL‐4, CD40L + IL‐6, CD40L + IL‐10, CD40L + IL‐17A, and CD40L + IL‐21). (f) Pie charts showing the distribution of B cell subsets in SP organoids treated with CD40L and various cytokine combinations. (g) Quantification of B cell numbers (CD19^+^) in HIS mouse SP organoids after cytokine treatment. (h) Quantification of B cell subsets (TR, Naive, Bmem, and PB) as a percentage of CD19^+^ cells in SP organoids treated with CD40L and cytokine combinations (*n =* 3). Statistical significance was calculated by one‐way ANOVA. *P* < 0.05 was shown. Data are presented as mean ± s.d.

Given the observed differentiation of Bmem cells in HIS mouse SP organoids, it was hypothesized that BCR stimulation alone, as the first signal, might be sufficient for B cell activation in vitro. Thus, we sought to directly supplement HIS mouse SP organoids with various cytokines and quantify B cell expansion and differentiation using flow cytometry. Among the cytokines tested (IL‐2, IL‐4, IL‐6, IL‐10, IL‐17, and IL‐21), only IL‐2 promoted B cell expansion (Figure 2a,b). However, this effect may be attributed to paracrine cytokine secretion from T cells within the organoid, as T cell expansion was also observed (Figure 2c). IL‐4 treatment led to a phenotypic change in Naive B cells, with a reduction in CD24 expression, suggesting further activation induced by IL‐4 treatment (Figure 2a). Both IL‐2, IL‐10, and IL‐21 promoted a small degree of PB differentiation (Figure 2b,d). These findings highlight that HIS mouse SP organoids provide a valuable platform for investigating the factors that drive B cell differentiation and demonstrate the effects of individual cytokines on B cells in this model.

During the T‐B cell interaction phase, second signals provided by T cells include not only soluble cytokines but also membrane‐bound molecules. One key interaction in this process is the CD40‐CD40L co‐stimulatory signal [17]. To further evaluate how second signal cytokines influence B cell activation in HIS mouse SP organoids, we tested various cytokines in the presence of soluble recombinant CD40L (Figure 2e,f). We found that the combination of CD40L and IL‐4 significantly amplified B cell expansion, increasing the B cell population approximately 16.8‐fold (Figure 2g). Additionally, this combination caused a more pronounced downregulation of CD24 expression compared to IL‐4 treatment alone, with a subset of cells acquiring a Bmem phenotype (CD38^−^) (Figure 2e). The combination of CD40L with IL‐10 (6.8%) and IL‐21 (12.9%) also promoted PB differentiation (Figure 2e–h). These results indicate that CD40L, in combination with specific cytokines, can significantly enhance B cell activation, expansion, and differentiation within HIS mouse SP organoids, further validating this system as a useful platform for screening second signal components that regulate human B cell responses.

**FIGURE 3:**
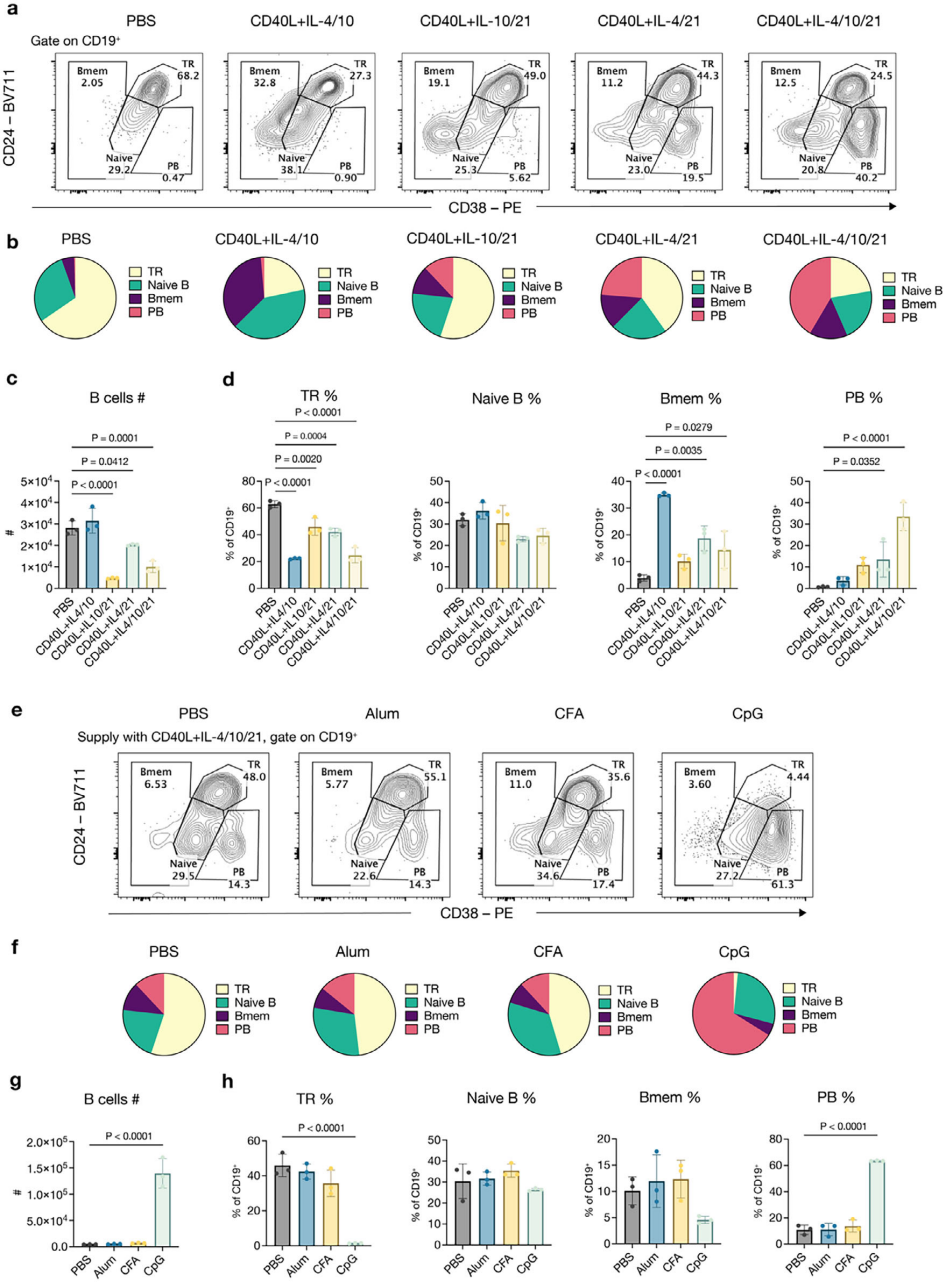
Maximizing B cell expansion and differentiation in HIS mouse SP organoids with cytokine and adjuvant combinations. (a) Flow cytometry analysis of B cell subsets (Naive, TR, Bmem, and PB) in HIS mouse SP organoids treated with combinations of CD40L and various cytokines (IL‐4/10, IL‐10/21, IL‐4/21, and IL‐4/10/21). The proportions of B cell subsets were measured based on CD19, CD24, and CD38 expression. (b) Pie charts illustrating the distribution of B cell subsets in SP organoids treated with different cytokine combinations (*n =* 3). (c) Quantification of B cell numbers (CD19^+^) in HIS mouse SP organoids after cytokine treatment. (d) Quantification of B cell subsets (TR, Naive, Bmem, and PB) as a percentage of CD19^+^ cells in SP organoids treated with different cytokine combinations. e) Flow cytometry analysis of B cell subsets in HIS mouse SP organoids treated with CD40L + IL‐4/10/21 and various adjuvants (Alum, CFA, and CpG). (f) Pie charts showing the distribution of B cell subsets in SP organoids treated with CD40L + IL‐4/10/21 and different adjuvants. (g) Quantification of B cell numbers (CD19^+^) in HIS mouse SP organoids after cytokine treatment. h) Quantification of B cell subsets (TR, Naive, Bmem, and PB) as a percentage of CD19^+^ cells in SP organoids treated with CD40L + IL‐4/10/21 and different adjuvants (*n =* 3). Statistical significance was calculated by one‐way ANOVA. *p* < 0.05 was shown. Data are presented as mean ± s.d.

Given the observed effects of CD40L, IL‐4, IL‐10, and IL‐21 on B cell expansion and differentiation in HIS mouse SP organoids, we further explored combinations of these cytokines and membrane proteins to maximize B cell proliferation and differentiation (Figure 3a,b). IL‐2 was excluded from our experiments, as it induced substantial T cell expansion, regardless of whether it was administered alone or in combination with CD40L (Figure S1a). Among the cytokine combinations tested (CD40L + IL‐4/10, CD40L + IL‐10/21, CD40L + IL‐4/21, and CD40L + IL‐4/10/21), we found that the combination of CD40L and IL‐4 is essential for B cell expansion (Figure 3c). Notably, the combination of CD40L + IL‐4/10/21 induced the most significant differentiation of B cells into PB (33.5%) and therefore was selected as the optimal second signal formula for B cell activation (Figure 3d).

To address whether effector B cell subsets accumulation in HIS mouse SP organoids reflects active differentiation rather than selective survival, we compared B cell subsets at D0 (pre‐culture) and D7 under PBS, CD40L+IL‐4, and CD40L+IL‐4/10/21 conditions, with Ki‐67 staining to assess proliferation and additional CD27 and BCL6 markers for further subset characterization (Figures S2a and S9a,b). In the PBS group at D7, the absolute number of Bmem cells and the proportion of Ki‐67^+^ Bmem were both higher than at D0, indicating that spontaneous Bmem accumulation during organoid culture results from active proliferation and differentiation (Figure S2b–e). CD40L+IL‐4 and CD40L+IL‐4/10/21 treatment further increased the absolute numbers of total B cells, Ki‐67^+^ B cells, and Ki‐67^+^ subsets, confirming that the second signals drive active B cell proliferation and differentiation in the organoid system (Figure S2b–e).

We further examined whether immune cell subsets within the organoids contribute to PC differentiation under second signal stimulation. After depletion of individual cell populations and culture with CD40L+IL‐4/10/21 for seven days, depletion of CD4^+^ T cells, CD4^+^ and CD8^+^ T cells, CD11b^+^ cells, and CD11c^+^ cells led to reduced PC numbers and proportions, while removal of CD8^+^ T cells or NK cells had minimal effects (Figure S3a,b). These results suggest that CD4^+^ T cells and myeloid cell populations within the organoids support PC differentiation in the presence of second signals.

### 2.3 Effects of Adjuvants on B Cell Responses in HIS Mice

In vaccine immunology, adjuvants stimulate innate immune cells to initiate immune responses, and certain adjuvants such as CpG and R848 can directly act on B cells through TLR7/9 receptors [34]. CpG ODN 2395 has demonstrated the ability to enhance antigen‐specific B cell responses in HIS mice in a previous study [31]. We hypothesized that CpG might amplify the effects triggered by the second signal. To test this hypothesis, we investigated the effects of three different adjuvants—Alum, complete Freund's adjuvant (CFA), and CpG (CpG ODN2395)—on B cell responses in HIS mouse SP organoids (Figure 3e,f). Under the CD40L + IL‐4/10/21 treatment condition, we observed that the addition of CpG significantly enhanced B cell expansion and led to a higher proportion of PB cells (Figure 3f). This suggests that CpG not only promotes B cell proliferation but also facilitates their differentiation into PB cells within the context of HIS mouse SP organoids (Figure 3g,h).

### 2.4 Innate Immune Signals Promote Antibody Class‐Switch in HIS Mouse SP Organoids

**FIGURE 4:**
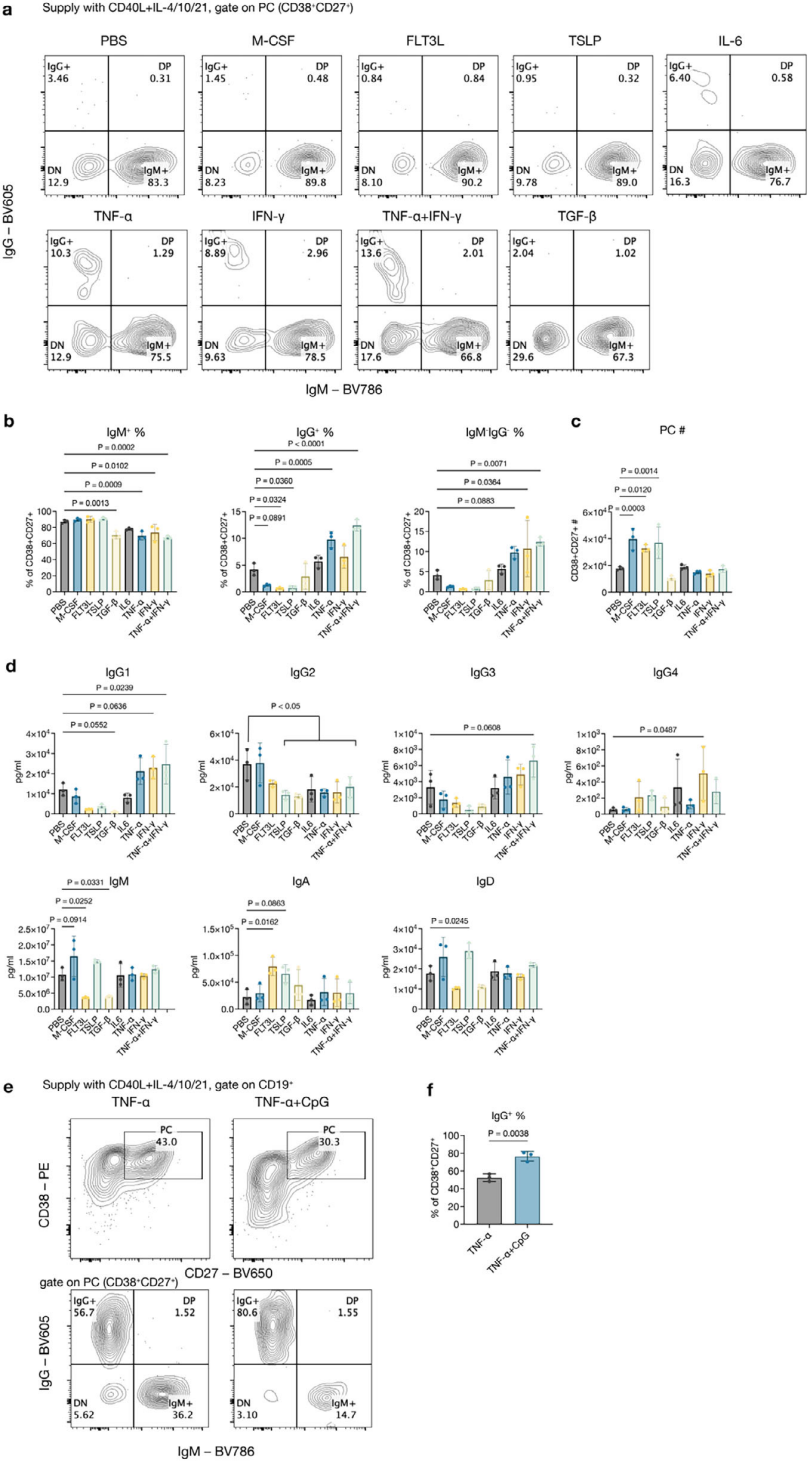
Innate immune signals promote antibody class‐switch in HIS mouse SP organoids. (a) Flow cytometry analysis of IgM and IgG expression in PC from HIS mouse SP organoids treated with different cytokines on top of CD40L+IL‐4/10/21. Representative plots are shown for PBS and cytokine treatment groups. PC were gated as CD38^+^CD27^+^ cells. (b) Quantification of the proportions of IgM^+^ and IgG^+^ cells within the PC population in organoids treated with the indicated cytokines. (c) Quantification of PC numbers in HIS mouse SP organoids treated with the indicated cytokines. (d) Quantification of antibody isotypes in the culture supernatants from HIS mouse SP organoids treated with the indicated cytokines. (e) Flow cytometry analysis of IgM and IgG expression in PC from HIS mouse SP organoids treated with TNF‐α alone or in combination with CpG on top of CD40L+IL‐4/10/21. Representative plots are shown. (f) Quantification of the proportion of IgG^+^ cells within the PC population under the indicated treatment conditions. Statistical significance was calculated by one‐way ANOVA. *p* < 0.05 was shown. Data are presented as mean ± s.d.

Antibody class‐switch is a key step in B cell responses. Although the second signal combination identified above promoted B cell expansion and differentiation, the resulting PB were predominantly IgM^+^, with 87.2% expressing IgM, indicating insufficient class‐switch under these culture conditions (Figure 4a,b). We therefore hypothesized that innate immune signals, in addition to CD4^+^ T cell‐derived signals, may also regulate class‐switch in human B cells. To test this, we screened additional cytokines in HIS mouse SP organoids on top of CD40L+IL‐4/10/21.

The screened signals fell into three categories. The first included M‐CSF, FLT3L, and TSLP, which primarily support the differentiation and maintenance of innate immune cells such as macrophages and DCs within the organoids. The second included TNF‐α and IL‐6 as directly acting inflammatory cytokines. The third included IFN‐γ and TGF‐β, which can also be secreted by different CD4+ T cell subsets. Compared to PBS controls, TNF‐α and TNF‐α+IFN‐γ treatment increased the proportion of IgG^+^ cells within the PC population, with TNF‐α+IFN‐γ showing the strongest effect, raising IgG^+^ PC from 4.1% to 12.4%. TNF‐α reached 9.8% (Figure 4a,b). Correspondingly, the proportion of IgM^+^ PC decreased in these groups, confirming that these inflammatory signals facilitated IgM‐to‐IgG switching (Figure 4b).

Beyond class‐switch, several factors also affected PC numbers. Compared to PBS controls, M‐CSF, FLT3L, and TSLP treatment significantly increased PC numbers to 3.98 × 10^4^, 3.28 × 10^4^, and 3.7 × 10^4^, respectively, versus 1.78 × 10^4^ in the PBS group (Figure 4c). These factors thus appeared to promote PC generation or survival rather than IgG switching.

We next quantified antibody isotypes in the culture supernatants. Different factors had distinct effects on antibody isotype secretion profiles (Figure 4d). Inflammatory signals, particularly TNF‐α, and TNF‐α+IFN‐γ, preferentially elevated IgG1 and IgG3 secretion but did not promote IgG2 production. In contrast, M‐CSF and TSLP treatment maintained high levels of IgM and IgD secretion, consistent with their ability to expand PC without inducing class‐switch. Interestingly, FLT3L suppressed IgM secretion but promoted IgA production. Taken together with the FACS data, TNF‐α emerged as candidate signals for promoting class‐switch.

Given that TNF‐α showed the strongest IgG‐promoting activity in the initial screen, we further tested its combination with other innate immune stimuli. Combining TNF‐α with CpG markedly enhanced class‐switch, raising the IgG^+^ PC proportion to 76.6%, significantly higher than TNF‐α alone (Figure 4e,f). This result indicates that TLR‐mediated innate immune activation synergizes with TNF‐α to drive IgG switching of human B cells in HIS mouse SP organoids.

In summary, organoid‐based screening revealed that different innate immune signals serve distinct functions in B cell responses. TNF‐α and TNF‐α+IFN‐γ primarily promote class‐switch, while M‐CSF, FLT3L, and TSLP preferentially expand PC numbers. Furthermore, CpG synergizes with TNF‐α to substantially increase the IgG^+^ PC proportion, providing a rationale for subsequent in vivo optimization of B cell responses.

### 2.5 Kinetics of B Cell Responses in HIS Mouse SP Organoids

**FIGURE 5:**
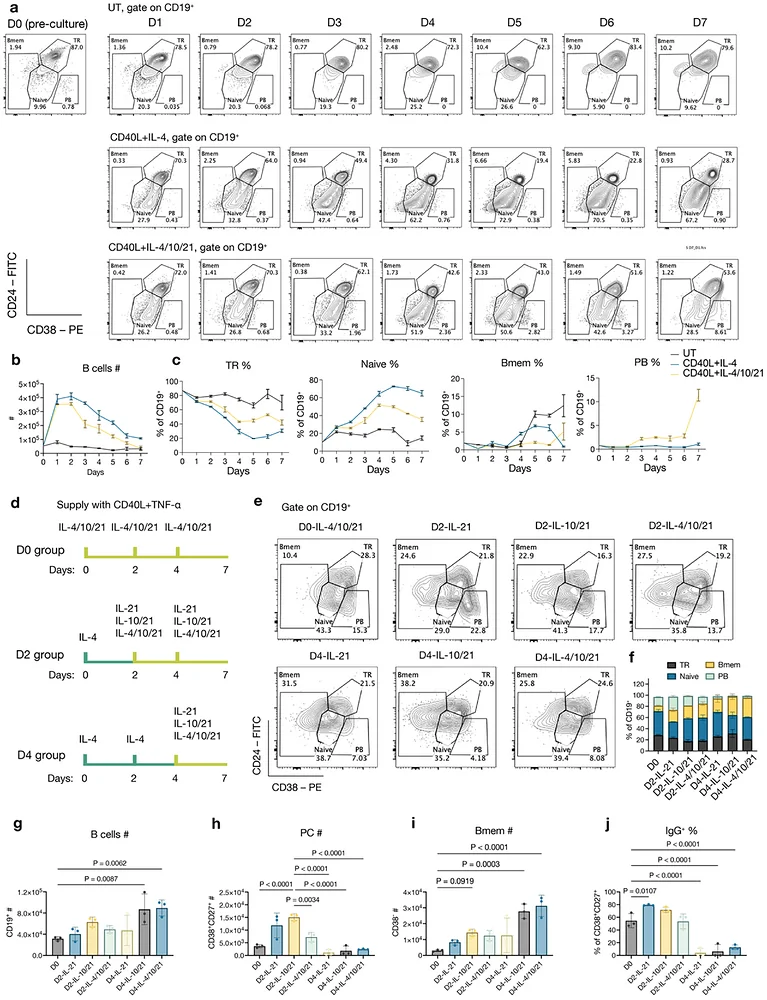
Kinetics of B cell responses and temporal optimization of expansion and differentiation signals in HIS mouse SP organoids. (a) Representative flow cytometry plots showing B cell subset composition (TR, Naive, Bmem, and PB) from D0 to D7 in HIS mouse SP organoids under three conditions: untreated (UT), CD40L+IL‐4, and CD40L+IL‐4/10/21. B cell subsets were gated on CD19^+^ cells and defined by CD24 and CD38 expression. (b) Quantification of total B cell numbers (CD19^+^) over time in UT, CD40L+IL‐4, and CD40L+IL‐4/10/21 treated organoids from D0 to D7. (c) Kinetics of B cell subset proportions (TR, Naive, Bmem, and PB as percentage of CD19^+^) from D0 to D7 under the three treatment conditions described in (a). (d) Schematic of the temporal signal separation experiment. All groups received CD40L+TNF‐α throughout the culture period. D0 group received IL‐4/10/21 from D0 to D7. D2 group received IL‐4 from D0 to D2, followed by a complete medium change to differentiation signals (IL‐21, IL‐10/21, or IL‐4/10/21) from D2 to D7. D4 group received IL‐4 from D0 to D4, followed by a medium change to the same differentiation signals from D4 to D7. (e) Representative flow cytometry plots of B cell subsets at D7 for the indicated treatment groups. B cell subsets were gated on CD19^+^ cells and defined by CD24 and CD38 expression. (f) Stacked bar graph showing the proportions of TR, Naive, Bmem, and PB within CD19^+^ B cells at D7 for all treatment groups. (g) Quantification of total B cell numbers (CD19^+^) at D7 across treatment groups. (h) Quantification of PC numbers (CD38^+^CD27^+^) at D7 across treatment groups. (i) Quantification of Bmem numbers (CD38^−^) at D7 across treatment groups. j) Quantification of IgG^+^ cell proportion within PC (CD38^+^CD27^+^) at D7 across treatment groups. Statistical significance was calculated by one‐way ANOVA. *p* < 0.05 was shown. Data are presented as mean ± s.d.

To better understand the kinetic profile of B cell responses in HIS mice and to inform the optimization of stimulation protocols, we cultured HIS mouse SP organoids under three conditions: untreated, CD40L+IL‐4, and CD40L+IL‐4/10/21, and analyzed B cell expansion and differentiation daily from D0 to D7 (Figure 5a).

Total B cell number analysis showed that both second signal‐treated groups underwent rapid early expansion, while B cell numbers in the untreated group declined steadily from D0 to D7, reflecting spontaneous apoptosis in the absence of survival signals (Figure 5b). Under CD40L+IL‐4 and CD40L+IL‐4/10/21 conditions, B cell numbers peaked at D2, reaching approximately 4.1 × 10^5^ and 3.6 × 10^5^, respectively, compared to only 5.3 × 10^4^ in the untreated group. After D2, B cell numbers gradually declined in both treated groups, though the CD40L+IL‐4 group maintained relatively higher cell numbers at later time points. In contrast, the CD40L+IL‐4/10/21 group declined more rapidly, suggesting that IL‐10 and IL‐21 promoted differentiation at the expense of a shortened expansion phase. The overall similarity in expansion kinetics between the two treated groups indicates that CD40L+IL‐4 was sufficient to provide the primary proliferative signal, with limited additional contribution from IL‐10 and IL‐21 to total B cell expansion.

In the untreated group, the TR proportion remained relatively stable from D0 to D5 (62%–80%), but spontaneous Bmem accumulation began at D4, with the Bmem proportion gradually increasing to 10% by D4‐D7 (Figure 5c).

In both second signal‐treated groups, the TR proportion declined from D1 onward, accompanied by a progressive increase in the Naive proportion, which reached 62.2%–73% (CD40L+IL‐4) and 48.5%‐52.5% (CD40L+IL‐4/10/21) by D4‐D5 (Figure 5a,c). This shift was associated with CD24 downregulation on Naive B cells following second signal treatment (Figure 5a), indicating that the second signal not only drove B cell expansion but also induced phenotypic maturation.

Regarding PB differentiation, the untreated group generated almost no detectable PB throughout the observation period. The CD40L+IL‐4 group also produced only minimal PB. In contrast, the CD40L+IL‐4/10/21 group displayed a biphasic PB differentiation pattern (Figure 5a,c): the first wave appeared at D2‐D3 (1.96%–2.36%), followed by a brief decline; the second wave began at D5 and continued to rise, reaching 8.61% at D7. This biphasic pattern may reflect sequential waves of plasmacell differentiation from B cells at different stages of activation. Given that total B cell expansion peaked at D2, the early PB may represent rapidly responding short‐lived plasmacells, while the later PB likely arose from B cells that underwent a more prolonged activation and differentiation process.

Overall, these kinetic data establish the temporal sequence from B cell expansion to differentiation in HIS mouse SP organoids. B cell expansion peaked at D2, spontaneous Bmem differentiation occurred preferentially in the absence of exogenous stimulation, and CD40L+IL‐4/10/21 favored PB differentiation with two distinct waves at D2 and D6. These findings provided the basis for determining the optimal timing of intervention in subsequent experiments.

### 2.6 Temporal Separation of Expansion and Differentiation Signals Optimizes B Cell Responses in HIS Mouse SP Organoids

The kinetic analysis described above revealed that B cell expansion peaked at D2, whereas PB differentiation occurred at later time points, suggesting that expansion and differentiation are driven by distinct signal requirements with a sequential temporal relationship. We therefore hypothesized that delivering expansion and differentiation signals separately over time may be more effective than providing all signals simultaneously from D0 (CD40L+IL‐4/10/21).

To test this, we split the second signal into two modules: CD40L+IL‐4 as the expansion signal and CD40L+IL‐10/21 as the differentiation signal. All groups were supplemented with TNF‐α to support class‐switch. The experimental design was as follows (Figure 5d): the D0 group received CD40L+IL‐4/10/21 from the start of culture, serving as the simultaneous signal control, the D2 group received IL‐4 from D0 to D2 for expansion, followed by a complete medium change to differentiation signals at D2; the D4 group extended the expansion phase to D4 before switching. For the differentiation phase, we further compared three signal compositions containing IL‐21 alone, IL‐10/21, or IL‐4/10/21, to evaluate the contribution of each cytokine to differentiation outcomes. All groups were harvested for analysis at D7.

FACS analysis revealed distinct changes in B cell subset composition in the D2 and D4 switch groups compared to the D0 group (Figure 5e,f). Total B cell numbers were highest in the D4 switch groups (9 × 10^4^), likely because the extended expansion phase (D0‐D4) allowed greater B cell accumulation (Figure 5g).

For PC (CD38^+^CD27^+^) differentiation, the D2 groups switching to IL‐10/21 yielded the highest PC numbers (1.5 × 10^4^), significantly exceeding both the D0 group (3.6 × 10^3^) and the D4 switch groups (2.4 × 10^3^) (Figure 5h). This indicates that switching to differentiation signals at the peak of B cell expansion (D2) most efficiently directed expanded B cells toward PC differentiation. By contrast, the D4 switch groups had higher total B cell numbers but lower PC output, suggesting that an excessively long expansion phase caused some B cells to miss the optimal differentiation window. Notably, regardless of whether the switch occurred at D2 or D4, differentiation signals containing only IL‐21 produced fewer PC than those containing IL‐10, further supporting the role of IL‐10 in promoting PC differentiation (Figure 5h).

For Bmem differentiation, the D4 switch groups (3.1 × 10^4^) generated significantly more Bmem than the D2 switch groups (1.4 × 10^4^) and the D0 group (3.1 × 10^3^) (Figure 5i), indicating that a longer expansion phase favored Bmem generation. This contrasted with the preference of PC differentiation for D2 switching, suggesting that the duration of the expansion phase may influence B cell fate decisions.

For class‐switch, the D2 group switching to IL‐21 showed the highest IgG^+^ % in PC (79.3%) proportion, significantly above the D0 (54.7%) simultaneous signal condition (Figure 5j). The D2 group switching to IL‐10/21 also exhibited a relatively high IgG^+^ proportion (71.3%). Among the D4 switch groups, the IL‐4/10/21 combination induced some degree of IgG switching (12.3%), though overall efficiency was lower than in the D2 switch groups.

Collectively, temporal separation of expansion and differentiation signals outperformed the simultaneous delivery of all signals. Switching to differentiation signals at D2 maximized PC output and IgG class‐switch, while extending the expansion phase to D4 favored Bmem generation. These organoid‐derived temporal optimization results provided a framework for designing cytokine administration timing in subsequent in vivo experiments.

### 2.7 Hydrodynamic Injection of Plasmids Encoding Second Signals Induces Non‐Specific B Cell Differentiation and Decreased Body Weight in HIS Mice

Hydrodynamic injection of plasmids encoding cytokines has been reported as a method to promote the activation and expansion of antigen specific B cell cells in HIS mice [28, 35, 36]. To assess whether supplying the second signals identified in our in vitro screening could also be effective in vivo, we injected 10 µg plasmids (using pLIVE vector) encoding human IL‐4, CD40L, or IL‐4, IL‐10, IL‐21, and CD40L into HIS mice via hydrodynamic injection, along with 40 µg of empty vector plasmids as a control (Figure S4a). Notably, the N‐terminal of CD40L was fused with a Gluc secretion signal peptide, and the ACRP30 collagen domain was added to enhance trimerization potential.

Seven days post‐injection, flow cytometry analysis revealed that the IL‐4/10/21/CD40L treatment group showed 15.9% PB cells in the SP, indicating significant B cell differentiation (Figure S4b–d). Moreover, serum antibody levels were significantly elevated in the IL‐4/10/21/CD40L group (Figure S2c). Additionally, we observed that both the IL‐4/CD40L and IL‐4/10/21/CD40L treatment groups exhibited CD24 downregulation on Naive B cells, consistent with the B cell phenotype observed in SP organoids treated with IL‐4 and CD40L (Figure S4e). However, a significant weight loss of 20.7% was observed in the IL4/10/21/CD40L treatment group by day 7 post‐injection (Figure S4f). Considering non‐specific B cell differentiation and a significant reduction in body weight, we conclude that hydrodynamic injection of plasmid encoding second signals is not suitable for activation of antigen‐specific B cell responses in HIS mice.

### 2.8 “Antigen Primed‐Second Signal Supplementation” Induces Antigen‐Specific B Cell Responses in HIS Mice

**FIGURE 6:**
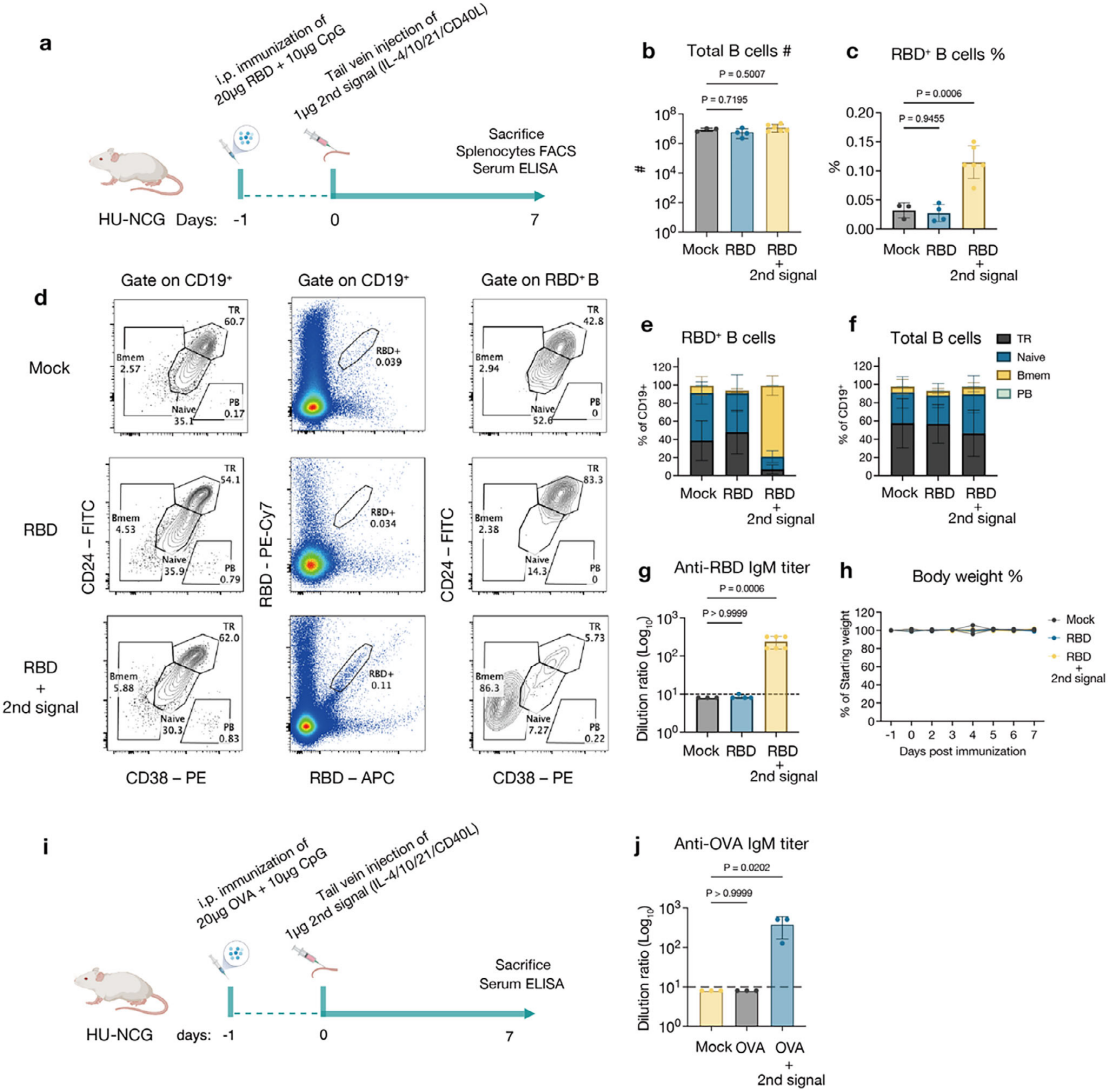
“Antigen‐primed second signal supplementation” induces antigen‐specific B cell responses in HIS mice. (a) Schematic of the experimental design. HU‐NCG mice were first immunized with 20 µg of SARS‐CoV‐2 RBD protein and 10 µg of CpG adjuvant via intraperitoneal (i.p.) injection 24 h prior to the administration of a second signal. Afterward, 1 µg each of IL‐4, IL‐10, IL‐21, and CD40L protein was administered via tail vein injection to stimulate B cell differentiation (Mock, *n =* 3; RBD+CPG, *n =* 4; RBD + 2nd signal, *n =* 6). Mice were sacrificed at 7 days post‐immunization (dpi) for flow cytometry and serum analysis. (b) Total B cell numbers in the SP of RBD‐immunized mice with or without the second signal. No significant difference was observed in total B cell numbers. (c) Percentage of RBD^+^ B cells in the SP at 7 dpi. A significant increase in the proportion of RBD‐specific B cells was observed in the second signal‐treated group. (d) Flow cytometry analysis of RBD^+^ B cell subsets from SP at 7 dpi. The percentage of TR, Naive, and Bmem B cells was assessed, showing a shift from TR and Naive phenotypes (CD38^+^CD24^−^) to Bmem phenotype (CD38^−^). (e) Quantification of RBD‐specific B cell subsets (Naive, TR, Bmem, and PB) as a percentage of CD19^+^ B cells. (f) Analysis of RBD‐specific B cell phenotypes in SP at 7 dpi, showing a predominant shift to Bmem cells in the second signal‐treated group. (g) Anti‐RBD IgM titers in serum measured by ELISA. The RBD + second signal group showed a significant increase in anti‐RBD IgM levels compared to the RBD + CpG‐only group. (h) Body weight changes in RBD‐immunized HIS mice. (i) Schematic of the experimental design for the OVA immunization model (*n =* 3). (j) Anti‐OVA IgM titers in serum measured by ELISA. The dashed line indicates the limit of detection (LOD). Undetectable values in ELISA were set to LOD – 0.2 log units to distinguish them. Statistical significance was calculated by unpaired t‐test. Statistical significance was calculated by one‐way ANOVA. Data are presented as mean ± s.d.

To accurately deliver artificial secondary signals to antigen‐specific B cells, we first activated these cells using cognate antigens to heighten their sensitivity to secondary signaling. We used the SARS‐CoV‐2 RBD protein, a well‐characterized antigen that effectively activates human B cell responses [37]. Additionally, we previously established a method to detect RBD‐specific responses using antigen tetramers [38]. As a proof‐of‐concept, we first immunized HIS mice intraperitoneally (i.p.) with 20 µg of RBD and 10 µg of CpG adjuvant, and delivered the second signal 24 hours later via tail vein injection of 1 µg each of IL‐4, IL‐10, IL‐21, and CD40L proteins (Figure 6a). The 1 µg per‐cytokine dose was chosen based on our previous experience that 5 µg of FLT3L protein administered i.v. was sufficient to promote human DC reconstitution in HIS mice [25], and was deliberately reduced to minimize non‐specific bystander activation while maintaining stimulatory capacity toward antigen‐primed B cells. At 7 days post‐immunization (dpi), flow cytometry analysis of HIS mouse SP revealed that while the overall B cell phenotype and numbers remained unchanged (8.9 × 10^6^ vs. 1.3 × 10^7^, *P* = 0.5), the proportion (0.032 vs. 0.115%) of RBD‐specific B cells (RBD^+^) were significantly increased in the second signal‐treated group (Figure 6b,c). Notably, analysis of RBD^+^ B cell phenotypes showed a marked shift from TR and Naive B cells (CD38^+^CD24^−^) to Bmem (CD38‐), indicating the successful differentiation of B cells (Figure 6d,f). Meanwhile, the overall B cell phenotype remained unchanged, indicating that this strategy does not lead to bystander B cell activation (Figure 6d).

Compared to mice that were only immunized with RBD and CpG, the addition of the second signal significantly enhanced the RBD‐specific IgM titers (Figure 6g). In contrast to hydrodynamic injection of plasmid DNA encoding second signals, which led to significant weight loss, mice treated with the protein form of the second signals showed stable body weight, suggesting that the tail vein injection of the second signal proteins was well‐tolerated in vivo (Figure 6h).

To assess the general applicability of this strategy to other antigens, we immunized HIS mice with OVA and CpG, followed by the second signal 24 h later. As in the RBD model, the addition of the second signal led to a significant increase in anti‐OVA antibody titers compared to the Mock and OVA + CpG‐only group (Figure 4i,j). In summary, these results demonstrate that the sequential administration of antigen sensitization followed by low‐dose second signal supplementation can effectively rescue antigen‐specific B cell responses in HIS mice, with applicability to different antigens such as RBD and OVA.

### 2.9 Temporal Separation of Expansion and Differentiation Signals Optimizes Antigen‐Specific B Cell Responses in HIS Mice

In the preceding experiments, a single dose of second signals promoted RBD‐specific B cell expansion and Bmem differentiation and increased anti‐RBD IgM titers, indicating insufficient B cell differentiation and class‐switch. Based on two key findings from organoid optimization, namely that TNF‐α promotes class‐switch (Figure 4) and that temporal separation of expansion and differentiation signals enhances B cell responses (Figure 5), we sought to validate these strategies in vivo.

**FIGURE 7:**
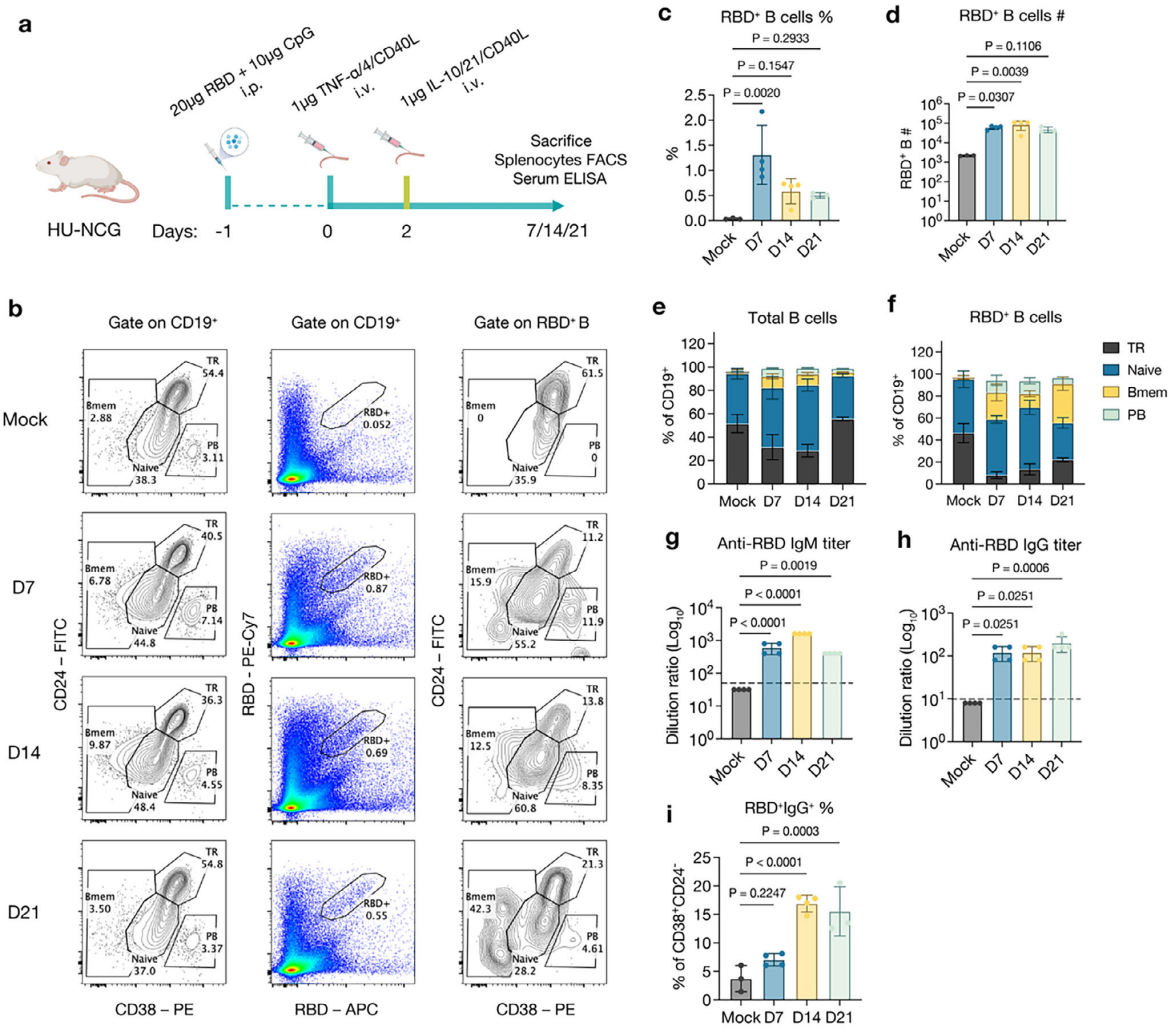
Temporal separation of expansion and differentiation signals combined with TNF‐α improves antigen‐specific IgG class‐switch in HIS mice. (a) Schematic of the experimental design. HU‐NCG mice were immunized with 20 µg of RBD protein and 10 µg of CpG adjuvant via i.p. injection at D‐1. At D0, an expansion signal consisting of 1 µg each of TNF‐α, IL‐4, and CD40L was administered via i.v. injection. At D2, a differentiation signal consisting of 1 µg each of IL‐10, IL‐21, and CD40L was administered via i.v. injection. Mice were sacrificed at D7, D14, or D21 for SP FACS analysis and serum ELISA (*n* = 3–4). (b) Representative flow cytometry plots showing total B cell subsets (left, gated on CD19^+^), RBD‐specific B cell frequency (middle, gated on CD19^+^), and RBD‐specific B cell subsets (right, gated on RBD^+^ B cells) in SP at the indicated time points. B cell subsets were defined by CD24 and CD38 expression. (c) Quantification of total B cell numbers (as percentage of CD19^+^ cells) in SP at the indicated time points. d) Quantification of RBD‐specific B cell frequency (as percentage of CD19^+^ cells) and absolute numbers in SP at the indicated time points. (e) Stacked bar graph showing the proportions of B cell subsets (TR, Naive, Bmem, and PB) within total CD19^+^ B cells at the indicated time points. (f) Stacked bar graph showing the proportions of B cell subsets (TR, Naive, Bmem, and PB) within RBD‐specific B cells at the indicated time points. (g) Anti‐RBD IgM titers in serum measured by ELISA at the indicated time points. The dashed line indicates the detection limit. (h) Anti‐RBD IgG titers in serum measured by ELISA at the indicated time points. The dashed line indicates the detection limit. (i) Quantification of IgG^+^ cell proportion within RBD‐specific PC (CD38^+^CD27^+^) at the indicated time points. Data are presented as mean ± s.d.

The immunization scheme was as follows (Figure 7a): HIS mice received 20 µg RBD + 10 µg CpG i.p. at D‐1 for antigen priming. At D0, an expansion signal mixture containing 1 µg each of TNF‐α, IL‐4, and CD40L was administered i.v. At D2, a second i.v. injection delivered the differentiation signal mixture containing 1 µg each of IL‐10, IL‐21, and CD40L. Mice were sacrificed at D7, D14, or D21 for SP B cell phenotyping and serum antibody analysis.

FACS analysis showed that the frequency of RBD‐specific B cells were significantly elevated at D7 (1.31%) and D14 (0.59%), and remained above Mock levels at D21 (0.5%) although the difference narrowed (Figure 7c). RBD^+^ B cell numbers were significantly increased in the D7 group (2.2 × 10^3^ vs. 6.1 × 10^4^) (Figure 7d). This kinetic pattern indicates that RBD‐specific B cells expanded during the early phase after immunization and gradually contracted thereafter.

In terms of B cell subset differentiation, total B cell phenotypes showed minimal variation across time points (Figure 7b,e). However, RBD‐specific B cells displayed clear differentiation progression (Figure 7b,f). By D14, Bmem further accumulated while PB proportions declined. At D21, PB proportions modestly rebounded, with Bmem maintained at elevated levels.

Serum antibody analysis revealed significantly elevated anti‐RBD IgM titers at D7 compared to Mock, and these levels were sustained through D14 and D21 (Figure 7g). More notably, in contrast to the single‐dose regimen where IgG was barely detectable, this optimized protocol yielded measurable anti‐RBD IgG titers (Figure 7h). This demonstrated that the inclusion of TNF‐α combined with temporal signal separation successfully promoted class‐switch of antigen‐specific B cells in vivo.

To further confirm that class‐switch occurred within antigen‐specific B cells, we analyzed the IgG^+^ proportion among RBD^+^ PB. The IgG^+^ frequency within RBD^+^ PB was significantly higher at D14 and D21 compared to Mock and D7 groups (Figure 7i), consistent with the kinetics of serum IgG elevation. This is consistent with the expectation that B cells require time to complete antibody switching following the delivery of differentiation signals at D2.

Together, these results demonstrate that translating the organoid‐derived TNF‐α class‐switch strategy and temporal signal separation protocol to the in vivo setting effectively induced expansion, Bmem differentiation, and IgG class‐switch of antigen‐specific B cells in HIS mice, resulting in detectable antigen‐specific IgG antibodies.

To evaluate whether T follicular helper (Tfh) cell responses accompanied B cell activation under our optimized protocol, we analyzed CXCR5^+^PD1^+^ Tfh cells within the CD3^+^CD4^+^ T cell compartment in SP at different time points (Figures S5a,b and S9c). At D7 and D14, Tfh frequencies remained comparable to Mock controls, suggesting that early B cell responses in this system likely proceed through an extrafollicular pathway. By D21, however, Tfh proportions were significantly elevated, raising the possibility that GC‐like reactions may develop at later time points and potentially contribute to the improved antibody affinity.

We also examined bone marrow (BM) B cells at D21 to assess whether antigen‐specific Bmem or PC had migrated to the BM (Figure S6). Although RBD‐specific B cell numbers and frequencies were modestly increased in the immunized group compared to Mock controls, the overall response in BM was weak, and RBD‐specific B cells remained predominantly at the immature/mature stage with minimal Bmem or PB differentiation. These findings suggest limited homing of antigen‐specific Bmem and PC to the BM under the current protocol.

To further confirm the generalizability of our optimized protocol, we immunized HIS mice with PE protein and CpG followed by temporal signal separation as described above (Figure S7a,b). The immunized group showed significantly elevated anti‐PE IgM and IgG titers compared to Mock controls, demonstrating that this strategy is applicable across different antigens beyond RBD and OVA.

### 2.10 Development of Fully Human Monoclonal Antibodies Using HIS Mice Supplied with Second Signals

**FIGURE 8:**
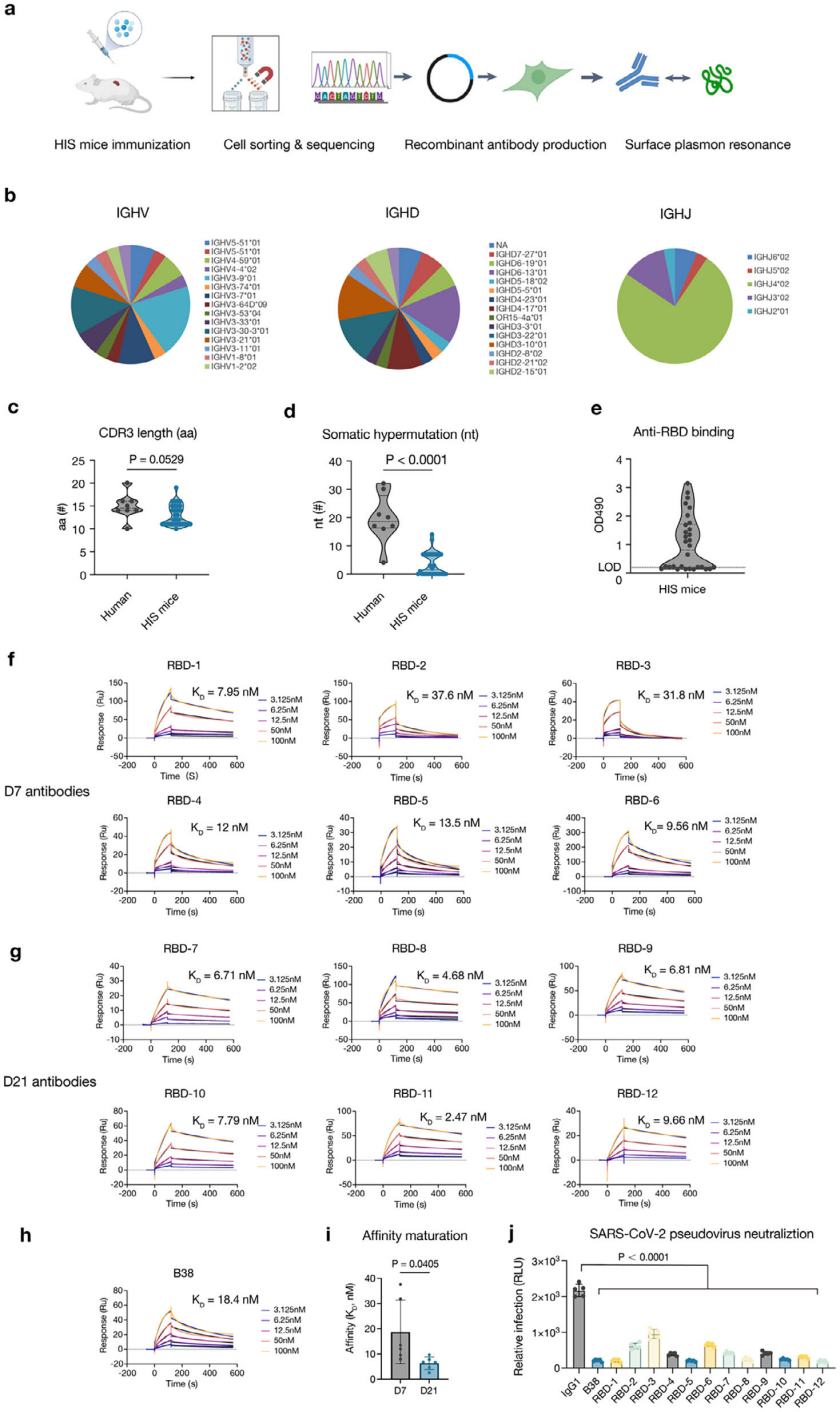
Development of fully human monoclonal antibodies using HIS mice and a second signal. (a) Schematic outlining the experimental process. HIS mice were immunized with RBD, and RBD^+^ B cells were sorted by flow cytometry at D7 or D21. The BCR regions from 30 individual B cells were sequenced. Recombinant antibodies were then expressed and purified. (b) Pie charts showing the diversity of V(D)J usage in the antibodies derived from HIS mice. (c) Comparison of CDR3 length between antibodies from HIS mice and human PBMC‐derived IgG^+^ B cells. (d) Comparison of somatic hypermutation (SHM) rates (number of mutations) between HIS mouse and human‐derived antibodies. (e) 30 recombinant antibodies derived from RBD^+^ B cells of HIS mice were evaluated by ELISA to assess their binding capacity to the RBD protein. The dashed line indicates the limit of detection (LOD). (f) Surface plasmon resonance (SPR) analysis of the affinity of recombinant HIS mouse antibodies to RBD at D7. (g) SPR analysis of the binding affinity of recombinant antibodies derived from HIS mice to RBD at D21. (h) SPR analysis of B38, a known human neutralizing antibody targeting RBD, shown as a positive control. (i) Comparison of RBD‐binding antibody affinities between D7 and D21. (j) Neutralizing activity of monoclonal antibodies against SARS‐CoV‐2 pseudoviruses. Neutralization assays were performed using pseudoviruse of the wild‐type SARS‐CoV‐2 on 293T‐hACE2 cells (*n* = 5). The known neutralizing antibody B38 was used as a positive control, and IgG1 served as an isotype control. Statistical significance was calculated by unpaired *t*‐test in (c, d, e, i) and one‐way ANOVA in (j). Data are presented as mean ± s.d.

To validate the utility of HIS mice in generating fully human monoclonal antibodies, we first sorted RBD‐specific B cells from the SP of HIS mice and sequenced the BCR regions of 30 individual B cells (Figure 8a). Upon analyzing the V(D)J sequences of these antibodies, we observed that HIS mice produced a diverse repertoire of antibody sequences, encompassing a variety of V(D)J recombination patterns (Figure 8b).

Comparing the sequences of antibodies cloned from HIS mice to those derived from human peripheral blood mononuclear cells IgG^+^ B cells, we found that the CDR3 length of HIS mouse‐derived antibodies were comparable to those from human IgG^+^ B cells. However, HIS mouse antibodies exhibited significantly fewer somatic hypermutations than their human counterparts (Figure 8c, d).

16 out of 30 recombinant antibodies derived from RBD^+^ B cells of HIS mice demonstrated in vitro RBD‐binding activity, as determined by ELISA (Figure 8e). Furthermore, surface plasmon resonance (SPR) analysis revealed that the six example recombinant antibodies bound to RBD with affinities ranging from 7.95 to 37.6 nm, indicating that these HIS mouse‐derived antibodies exhibit strong binding capabilities, with dissociation constants (K_D_) in the 10^−9^ to 10^−8^ m range (Figure 8f).

To further assess whether the optimized immunization protocol with temporal signal separation (Figure 7) could improve antibody quality, we sorted RBD‐specific B cells from HIS mice at D21 and performed BCR sequencing and recombinant antibody expression as described above. SPR analysis of six representative D21‐derived antibodies (RBD‐7 to RBD‐12) revealed K_D_ values ranging from 2.47 to 9.66 nm (Figure 8g), with several clones exhibiting higher affinities than the D7‐derived antibodies (Figure 8f). As a reference, the known human neutralizing antibody B38 showed a K_D_ of 18.4 nm under the same assay conditions (Figure 8h). Notably, the median affinity of D21 antibodies was higher than that of D7 antibodies (Figure 8i), suggesting that prolonged in vivo exposure following the optimized signal delivery may facilitate affinity maturation of antigen‐specific B cells in HIS mice.

We next evaluated the neutralizing capacity of these antibodies using a pseudovirus neutralization assay. Among the twelve recombinant antibodies tested, multiple clones from both D7 and D21 demonstrated potent neutralizing activity against SARS‐CoV‐2 WT pseudovirus, with several clones showing neutralization comparable to or exceeding that of B38 (Figure 8j).

Taken together, these data demonstrate that the optimized immunization and second signal delivery protocol can yield fully human monoclonal antibodies from HIS mice with high binding affinity and functional neutralizing activity against SARS‐CoV‐2 pseudovirus, further supporting the translational potential of this platform for therapeutic antibody discovery.

## 3 Discussion

This study addresses the longstanding issue of B cell dysfunction in HIS mice by proposing and validating a vaccination strategy termed “antigen‐primed second signal supplementation.” Through the establishment of a SP organoid screening platform, we identified IL‐4, IL‐10, IL‐21, and CD40L as components that promote B cell expansion and differentiation. Further organoid‐based screening revealed that innate immune signals, particularly TNF‐α and CpG, synergize to promote IgG class‐switch, and that temporal separation of expansion and differentiation signals enhances B cell output. Translating these findings in vivo, we demonstrated that sequential delivery of expansion signals (TNF‐α, IL‐4, CD40L) followed by differentiation signals (IL‐10, IL‐21, CD40L) after antigen priming successfully induced antigen‐specific B cell expansion, differentiation, IgG class‐switch, and the generation of antigen‐specific IgG antibodies in HIS mice. Moreover, RBD‐specific B cells sorted from immunized mice yielded fully human monoclonal antibodies with nanomolar binding affinity and neutralizing activity against SARS‐CoV‐2 pseudovirus. These findings provide a practical solution to bypass T cell dependency and directly support antigen‐specific B cell responses in HIS mice.

The HIS mouse SP organoid system we developed offers a controllable in vitro environment that maintains the integrity of diverse human immune cell populations, making it a tool for interrogating B cell functions. The SP organoid in this study refers to a multicellular reaggregation culture used as a signal screening platform, not a system intended to reconstruct lymphoid tissue architecture. Compared to MLN, Naive B cells in SP organoids remain stable without additional stimuli, and exhibit approximately 20% differentiation into Bmem, suggesting moderate BCR signaling activation within the culture, presumably reacting to the bovine serum. This feature makes the system a viable platform for screening second signal components without additional BCR stimulation, such as anti‐IgM antibodies. Furthermore, this platform reduces the cost of research as traditional in vivo screening methods require a large cohort of animals, whereas the organoid system can perform multi‐factor screening using a small number of HIS mice. Each HIS mouse provides approximately 10^7^ SP cells in our observation, sufficient for at least 50 organoid cultures. This organoid system not only enhances screening efficiency but also reduces animal use, adhering to the 3Rs principle (Replacement, Reduction, and Refinement). The system eliminates confounding effects from variations in hematopoietic stem cell genetic backgrounds and HIS mouse humanization levels, allowing researchers to focus on specific factors' impact on immune cells proliferation, differentiation, and signal transduction.

Our results support the hypothesis that the functional deficiency of B cells in HIS mice arises from the lack of T cell‐mediated second signals. In healthy human and mouse immune systems, antigen‐specific B cells, after receiving the first signal via BCR, require interaction with activated CD4^+^ T cells to obtain the second signal, including cytokine stimulation and co‐stimulatory signals via membrane‐bound receptors [13, 39]. However, in HIS mice, the activation of human T cells is impaired due to the inadequate DC population, which together hinder CD4^+^ T cells from effectively providing secondary signal support to B cells [6, 40]. The combination of IL‐4, IL‐10, IL‐21, and CD40L we identified may effectively simulate the complex signals provided by T cells by activating multiple signaling pathways, supposedly including JAK‐STAT, NF‐κB, and MAPK [14, 15, 16, 18]. In particular, IL‐4, in the presence of CD40L, promotes B cell expansion, while IL‐10 and IL‐21 drive PB differentiation.

Our organoid screening also uncovered a previously underappreciated role for innate immune signals in regulating antibody class‐switch in HIS mouse B cells. The initial second signal combination (CD40L+IL‐4/10/21) promoted robust B cell expansion and PB differentiation, but the resulting PB were predominantly IgM^+^, with limited IgG switching. Screening additional factors on this background revealed that TNF‐α, an inflammatory cytokine typically produced by innate immune cells, promoted IgM‐to‐IgG switching. CpG synergized with TNF‐α to raise the IgG^+^ PC proportion to over 80%, far exceeding either factor alone. This synergy between TLR9 activation and TNF‐α signaling suggests that innate immune activation provides signals that complement T cell‐derived factors in driving class‐switch. The incomplete class‐switch observed in vivo without TNF‐α, and the successful induction of IgG responses after incorporating TNF‐α into the immunization protocol, further validate these organoid‐derived findings and highlight the importance of innate immune signals in the overall B cell response.

An important insight from our organoid‐based kinetic and combinatorial analyses is that the signals governing B cell expansion and differentiation are temporally distinct. In physiological immune responses, CD4^+^ T cells initially provide proliferative signals dominated by CD40L and IL‐4 during early T‐B interactions, while cytokines such as IL‐10 and IL‐21 become predominant at later stages to drive plasmacell differentiation and class‐switch [39, 41]. Our organoid data recapitulated this principle: B cell expansion peaked at D2, while PB differentiation occurred at later time points with a biphasic pattern. Delivering all signals simultaneously from D0 was less effective than separating the expansion phase (CD40L+IL‐4, D0‐D2) from the differentiation phase (IL‐10/21, D2 onward). The duration of the expansion phase also influenced B cell fate: switching at D2 favored PB differentiation and class‐switch, while extending expansion to D4 preferentially generated Bmem. These observations suggest that the timing of signal delivery can be adjusted to bias B cell responses toward different effector outcomes depending on the experimental objective.

Compared to previous approaches aimed at improving T cell function in HIS mice through genetic modifications or complex humanization strategies [8, 21, 22], our artificial second signal approach is technically simple, easily standardized, and can be directly applied to existing HIS mouse strains without additional genetic engineering. Moreover, our strategy adopts a “workaround” approach, bypassing T cell dysfunction and directly providing the second signal required by antigen‐primed B cells.

To improve B cell responses in HIS mice by enhancing antigen presentation, previous studies have promoted myeloid‐cell development through hydrodynamic injection of GM‐CSF and IL‐4 or engineered antigens to more effectively target APCs [28, 42]. While these approaches can enhance antigen‐specific B cell responses, their broader application is limited by systemic inflammation, complex antigen design, and relatively long experimental timelines. Other approaches involve genetic modification of immunodeficient mice. For example, tissue specific expression of murine TSLP in BRGS mice has been shown to promote lymph node development and enhance B cell responses [24]. Another strategy involves the generation of hIL‐6 knock‐in mice to promote B cell maturation and differentiation [26]. Although an increased frequency of class‐switched B cells was observed, there remains insufficient evidence demonstrating antigen‐specific B cell expansion. In contrast, our approach provides a simpler and more practical method for enhancing antigen‐specific B cell responses in HIS mice, while avoiding systemic inflammation and complex antigen engineering. Importantly, this strategy is also applicable across multiple strains of humanized mice.

The choice of delivery strategy is a key technical challenge for in vivo application of artificial second signals. Hydrodynamic injection of cytokine‐encoding plasmids elevated serum antibody levels but caused non‐specific B cell activation, reduced SP B cell numbers, and systemic inflammation [42], likely due to prolonged cytokine expression that diverges from the short duration of physiological CD4^+^ T cell–B cell interactions during the extra‐follicular response [12, 43]. In contrast, tail vein injection of low‐dose recombinant proteins selectively enhanced the response of antigen‐primed B cells without disrupting overall B cell homeostasis. By delivering the second signal 24 h after antigen priming, our strategy preferentially activates antigen‐specific B cells while limiting bystander effects. Further optimization of dose gradients and targeted delivery systems may improve specificity and signal duration [44, 45, 46].

When antigen‐specific B cell responses were induced in HIS mice via artificial second signals, we assessed the potential of using HIS mice as a source of fully human monoclonal antibodies. We found that some recombinant antibodies exhibited high nanomolar affinity and neutralization activity, confirming that HIS mice can serve as a source for fully human monoclonal antibodies. The antibodies recovered from HIS mice can serve as lead candidates for further in vitro affinity maturation through established engineering approaches such as CDR mutagenesis and phage/yeast display. For vaccine evaluation purposes, the binding affinity and neutralizing activity observed here are sufficient to assess immunogenicity and epitope targeting. Additionally, the concept of artificial second signals may offer new therapeutic possibilities for immune deficiencies caused by T cell dysfunction, particularly those leading to humoral immune deficiency syndromes [43]. Furthermore, in the field of vaccine design, our findings suggest that a rational combination of antigens, adjuvants, and cytokines could optimize vaccine efficacy, which may be particularly important for elderly or immunocompromised populations [44, 45]. This strategy may also enhance immunization protocols for difficult‐to‐treat pathogens or cancer vaccines by improving the initiation of antigen‐specific B cell responses.

We note that the HIS mouse platform described here is best suited as an early discovery and lead‐identification tool for generating fully human antibody candidates, rather than a full surrogate for human GC maturation. Antibodies recovered from this system can serve as starting points for further affinity optimization through in vitro engineering approaches. The current limitation of this platform is the low level of SHM observed in HIS mouse‐derived antibodies compared to antibodies from human donors, likely reflecting the absence of structured GC reactions in HIS mice. The reason for this phenomenon could be that, despite the restoration of B cell responses through second signal supplementation, the absence of FDC and delayed differentiation of Tfh until D21, leading to an inadequate long‐term GC reaction. Nevertheless, the recovered antibodies exhibited nanomolar binding affinities and functional neutralizing activity. This observation is consistent with recent findings on SARS‐CoV‐2 neutralizing antibodies from human donors, where potent RBD‐targeting clones carry minimal somatic mutations, reflecting the inherent structural compatibility of certain germline genes such as IGHV3‐53 for RBD recognition [46]. In our model, antigen priming followed by second signal delivery may preferentially expand clones with higher intrinsic germline affinity, and the improved median affinity at D21 compared to D7 further supports ongoing clonal selection over time. However, RBD represents a strongly immunogenic antigen with a high frequency of germline‐reactive precursors in the human naive repertoire. Whether this platform can generate comparably high‐affinity antibodies against weaker immunogens that require extensive SHM‐driven affinity maturation remains to be determined. Further studies using a broader range of antigens will be required to address this question.

In conclusion, this study developed a SP organoid screening platform that enabled cost‐efficient identification of factors influencing B cell responses in HIS mice, including second signal cytokines, innate immune regulators of class‐switch, and the temporal sequence of signal delivery. By translating these organoid‐derived insights into an optimized in vivo immunization strategy combining antigen priming with sequential delivery of expansion and differentiation signals, we overcame barriers to antigen‐specific B cell responses in HIS mice, including insufficient expansion, lack of class‐switch, and poor effector differentiation. The resulting platform supports vaccine evaluation, fully human antibody discovery, and mechanistic studies of human B cell responses.

## 4 Materials and Methods

### 4.1 Study Design

All procedures for immunization, as well as the establishment and analysis of HIS mice, were reviewed and approved by the Institutional Animal Care and Use Committee (IACUC) of Nanjing University (Approval number: LY‐01). Sample sizes were not predetermined using statistical power calculations. Mice of comparable strain, age, and body weight were randomly allocated into experimental groups, and animals in the same treatment arm were administered reagents prepared from identical stock solutions.

### 4.2 Statistics and Reproducibility

Statistical analyses were conducted using GraphPad Prism 9.0 (GraphPad Software, San Diego, CA, USA). Normality of data distributions was assessed with the Shapiro–Wilk test. For datasets meeting the assumption of normality, unpaired two‐tailed t‐tests were applied for pairwise comparisons, and one‐way ANOVA followed by Dunnett's post‐hoc test was used for analyses involving more than two groups. For data not conforming to normal distribution, the Mann–Whitney U test was employed for two‐group comparisons, while the Kruskal–Wallis test with Dunn's multiple comparison test was used when analyzing more than two groups. Statistical significance was set at *p* < 0.05. Each group contained a minimum of three independent biological replicates.

### 4.3 Establishment of Splenic Organoid Cultures from HIS Mice

To reduce the cost of HIS mouse library screening and to model B cell activation ex vivo, we established splenic organoid cultures from HIS mice. Splenocytes and MLN cells were isolated from 10–20‐week‐old HU‐NCG mice, passed through a 100‐µm nylon strainer, washed, and resuspended in complete RPMI medium (RPMI 1640 supplemented with 10% FBS, 2 mm L‐glutamine, 1× nonessential amino acids, 1 mm sodium pyruvate, 1× penicillin–streptomycin). For SP sample, erythrocytes were removed with Red Blood Cell Lysing Buffer (Sigma, R7757). Cells were plated at 2 × 10^5^ per well in permeable membrane plates (0.4‐µm pore size, 24 well, Corning) with 200 µL of cell suspension in the upper chamber and complete medium in the lower chamber. Cultures were maintained at 37°C with 5% CO_2_. After 7 days, B cell phenotypes were analyzed by flow cytometry (Figure S9d). In this study, we used HIS mice generated from HSCs derived from three different donors. Within each experimental comparison, all samples were obtained from HIS mice reconstituted with HSCs from the same donor, thereby minimizing donor related variability within each dataset.

### 4.4 Magnetic Bead‐Based Depletion of Immune Cell Subsets from HIS Mouse Splenocytes

HIS mouse SP cells were prepared as single‐cell suspensions and resuspended in MACS buffer (PBS containing 0.5% BSA and 2 mm EDTA). Cells were incubated with biotin‐conjugated antibodies against the target subsets for 15 min at 4°C, including anti‐human CD4 (BioLegend, 300504), CD8a (BioLegend, 301004), CD11b (BioLegend, 301304), CD56 (BioLegend, 318320), CD11c (eBioscience, 13‐0116‐82), and anti‐mouse CD45 (BioLegend, 103104). After washing, cells were incubated with anti‐biotin magnetic beads (BeaverBio) for 15 min at 4°C following the manufacturer's instructions. Labeled cells were depleted using a magnetic separator, and the unbound fraction was collected. Unbound fraction cells were then plated on Transwell membranes at 2 × 10^5^ cells per well and cultured for seven days.

### 4.5 Cytokine Stimulation and Second‐Signal Testing

To identify candidate second signals for B cell activation, recombinant human cytokines (TNF‐α, GM‐CSF, M‐CSF, TSLP, FLT3L, IL‐2, IL‐4, IL‐6, IL‐10, IL‐17, IL‐21; PeproTech) were added to cultures at 10 ng/mL concentrations. CD40L (100 ng/mL, PeproTech) was used to mimic T cell help, alone or in combination with cytokines. B cell proliferation and differentiation were quantified by flow cytometry after 7 days.

### 4.6 Adjuvant Testing in Splenic Organoids

To evaluate the impact of adjuvants on B cell responses in SP organoids, cultures were stimulated with CD40L plus cytokines (IL‐4/10/21) in the presence or absence of adjuvants. CpG ODN2395 (20 ng/mL, InvivoGen) was dissolved in PBS and added directly to the culture medium. For Alum (20 µL/well, InvivoGen), the aluminum hydroxide gel suspension was vortexed thoroughly before addition to ensure even distribution in the culture well. For CFA (20 µL/well, Sigma), the oil‐in‐water emulsion was first vortexed vigorously, then added to the lower chamber of the Transwell system to avoid direct contact with the cell layer on the permeable membrane, as CFA is not miscible with aqueous culture medium. This configuration allowed the immunostimulatory components of CFA (primarily heat‐killed mycobacteria) to interact with cells through the permeable support while minimizing physical disruption of the organoid structure by the oil phase. An equal volume of PBS was added to control wells. B cell expansion and PB differentiation were assessed by FACS after 7 days of culture.

### 4.7 Human Immune System Mice

All mice were maintained under specific pathogen‐free (SPF) conditions in facilities accredited by the Institutional Animal Care and Use Committee (IACUC) at the Model Animal Research Center, Nanjing University (AP# LY‐01). NOD‐*Prkdc* ^scid^ *Il2Rγc* ^−/−^ (NCG, T001475) were obtained from GemPharmatech. Human immune system (HIS) mice were generated using NCG hosts as previously described [35]. Briefly, newborn pups (4–6 days old) received intrahepatic injections of 5 × 10^4^ CD34^+^ human fetal liver hematopoietic stem cells. 10 weeks later, 50 µL of peripheral blood was collected from each animal to assess human hematopoietic reconstitution (Figure S9e). Mice with human CD45^+^ cell counts exceeding 1 × 10^5^/mL were used for subsequent experiments.

### 4.8 Immunization

For i.p. immunization, antigens and 10 µg CpG ODN 2395 (InvivoGen, tlrl‐2395) were diluted in PBS using a 25 G syringe. The vaccine formulation was administered into the peritoneal cavity with a 27 G insulin syringe.

### 4.9 Hydrodynamic Injection

To evaluate the in vivo delivery of second signals via sustained gene expression, HIS mice received hydrodynamic tail vein injections of plasmid DNA encoding cytokines using the pLIVE vector (Mirus Bio), a vector designed for prolonged hepatic transgene expression driven by the murine albumin promoter. Control mice received 50 µg of empty pLIVE vector. The CD40L construct was modified by replacing the native N‐terminal cytoplasmic and transmembrane domains with a Gaussia luciferase (Gluc) secretion signal peptide to enable secretion, and the ACRP30 collagen domain was fused downstream of the signal peptide to promote trimerization of the soluble CD40L. All plasmid DNA was diluted in sterile saline to a final volume equivalent to 10% of mouse body weight and injected via the tail vein within 5–8 s to achieve hydrodynamic delivery to the liver. Mice were weighed daily and monitored for signs of distress. SP cells were harvested at 7 days post‐injection for FACS analysis, and serum was collected for antibody measurement by ELISA.

### 4.10 Tissue Processing and Flow Cytometry

Splenic and lymph node suspensions were prepared by mechanical dissociation through 100‐µm cell strainers. Following dissociation, single‐cell suspensions were obtained, erythrocytes were removed with Red Blood Cell Lysing Buffer (Sigma, R7757), and cells were resuspended in FACS buffer for staining. Flow cytometric data were collected on an Agilent NovoCyte Penteon cytometer equipped with five lasers (405 nm violet, 488 nm blue, 561 nm yellow, 637 nm red, and 349 nm ultraviolet). Antibodies secreted by B cells in the organoids were measured from supernatants collected after 7 days of culture. Detection was performed using the Human Ig Isotyping Panel (8‐plex) (BioLegend, 740638) according to the manufacturer's instructions. FACS antibodies include CD45‐BV711 (BioLegend, 304050, 1/100), CD45‐BUV395 (eBioscience, 363‐0459‐42, 1/100), mCD45‐VioGreen (MB, 130‐110‐665, 1/100), mCD45‐APC (BioLegend, 103112, 1/100), CD3‐APC‐Cy7 (BioLegend, 300426, 1/100), CD4‐PE (BioLegend, 300508, 1/100), CD19‐BUV395 (BD Biosciences, 363‐0459‐42, 1/100), CD19‐PE‐CF594 (BD Biosciences, 562294, 1/100), CD38‐PE (BioLegend, 303506, 1/100), CD24‐FITC (BioLegend, 311103, 1/100), CD24‐BV711 (BioLegend, 311136, 1/100), CD27‐BV650 (BD Biosciences, 563228, 1/100), CXCR5‐PE‐CF594 (BioLegend, 356928, 1/100), PD1‐BV786 (BioLegend, 329930, 1/100) IgD‐BV421 (BioLegend, 348225, 1/100), Ki‐67‐BV421 (BioLegend, 350505, 1/100), BCL6‐PE‐Cy7 (BioLegend, 358511, 1/100), IgM‐BV786 (BioLegend, B283254, 1/100) and IgG‐BV605 (BD Biosciences, 563246, 1/100).

### 4.11 RBD‐tetramer for Detecting RBD‐Specific B Cells

To assess B cell responses targeting RBD, the recombinant RBD protein (aa 319–591) was biotinylated using the EZ‐Link Sulfo‐NHS‐Biotin kit (Thermo Scientific, 21425) according to the manufacturer's protocol. The biotinylated RBD was then incubated with streptavidin conjugates (FITC (BioLegend, 405202), APC (BioLegend, 405207), or PE‐Cy7 (BioLegend, 405206)) at a 4:1 molar ratio for 30 min at room temperature to generate fluorescently labeled RBD tetramers. For flow cytometric analysis, single‐cell suspensions prepared from lymph nodes or lung tissues were stained with 1 pm RBD tetramer in 50 µL of FACS buffer (PBS containing 2% FBS, 2 mM EDTA, and 1% penicillin‐streptomycin) (Figure S9f).

### 4.12 Enzyme‐Linked Immunosorbent Assay

ELISA was performed to quantify antigen‐specific and total human immunoglobulin levels in serum samples. Blood samples were centrifuged at 3000 rpm for 10 min at 4°C after coagulation at room temperature for 2 h.

For antigen‐specific ELISA, flat‐bottom 96‐well ELISA plates (NEST, 514201) were coated with recombinant SARS‐CoV‐2 RBD protein (25 ng/100 µL/well) overnight at 4°C, blocked with 2% BSA in PBST, and incubated with serial dilutions of serum samples. Bound antibodies were detected with Peroxidase AffiniPure Goat Anti‐Human IgG (H+L) (Jackson ImmunoResearch, 109‐035‐003), followed by TMB substrate development (Sigma, 860336; 50 µL/well) for 10 min. The reactions were stopped with 50 µL/well of 1.0 M H_2_SO_4_ stop solution, and absorbance was measured at 490 nm.

For total immunoglobulin quantification, ELISA plates were first coated with capture antibodies (Goat anti‐human IgH antibody, Jackson ImmunoResearch), diluted in PBS, and incubated overnight at 4°C. After washing and blocking with 2% BSA in PBST, serum samples were added to the wells and incubated at 37°C for 1 h. Bound antibodies were detected with HRP‐conjugated goat anti‐human IgM or goat anti‐human IgG, respectively, followed by TMB substrate development and absorbance measurement as described above. Concentrations were determined using standard curves generated from purified human IgM or IgG proteins.

For antigen‐specific assays, endpoint titers were defined as the highest serum dilution yielding an OD490 value above the cut‐off (mean OD of negative control sera + SD). For total immunoglobulins, concentrations were interpolated from the standard curves. All samples were measured in duplicate, and raw OD values were background‐corrected by subtracting the mean OD of blank wells and accounting for background signal from negative control sera.

### 4.13 Pseudovirus Neutralization Assay

For pseudovirus generation, 293T cells were transfected with psPAX2, pLKO1‐Luciferase‐P2A‐GFP, and pcDNA3.1‐Spike (WT or Delta strain) using PEI (Polysciences, 23966). Pseudovirus‐containing supernatants were collected 48 h after transfection. For neutralization assays, 50 µL of pseudovirus (2 × 10^5^ TFU/mL) was mixed with 50 µL of serially diluted antibody and incubated at 37°C for 30 min before addition to 293T‐hACE2 cells (MOI = 1). Luciferase activity was measured using the Firefly Glo Luciferase Reporter Gene Assay Kit (YEASEN, 11404ES60) according to the manufacturer's instructions. The 293T‐hACE2 cell line used in the neutralization assay was generated by transducing 293T cells with a lentivirus encoding human ACE2 and transmembrane serine protease 2.

### 4.14 Index Sorting of RBD‐Specific B Cells and Antibody Cloning

Single‐cell suspensions were prepared from the SP of NCG humanized mice (HU‐NCG) as described above. Cells were stained with anti‐human CD45, anti‐human CD19, RBD‐PE‐Cy7, and RBD‐APC to identify RBD‐specific B cells. Index sorting was performed using a BD FACS Aria III cell sorter, gating on CD45^+^CD19^+^RBD‐PE‐Cf5947^+^RBD‐APC^+^ populations. Sorted single B cells were directly collected into lysis buffer for RNA extraction with RNeasy Mini Kit (QIAGEN, 74104). Total RNA was reverse‐transcribed into cDNA and IgH and IgL chain variable regions were amplified using a universal primer set covering human V gene families [46, 47]. Amplified antibody fragments were cloned into the expression vectors pFUSE‐IgG1 and pFUSE‐IgL (InvivoGen) for recombinant antibody expression.

### 4.15 Antibody Expression and Purification

Expression plasmids encoding the heavy and light chains of antibodies were co‐transfected into 293F suspension cells (OPM bioscience) at a 1:1 molar ratio using polyethyleneimine (PEI; YEASEN) as the transfection reagent, following the manufacturer's protocol. Cells were maintained in serum‐free medium under shaking culture conditions (37°C, 5% CO_2_, 150 rpm). After 6 days, culture supernatants were harvested by centrifugation at 4 000 × g for 10 min and filtered through 0.22‐µm membranes. Antibodies were purified from the supernatant using protein A magnetic beads (BeaverBio). Bound antibodies were eluted with acidic elution buffer and immediately neutralized with Tris‐HCl (pH 8.0). Purified antibodies were buffer‐exchanged into PBS using centrifugal filters and quantified by absorbance at 280 nm.

### 4.16 Surface Plasmon Resonance Assay

Binding kinetics between antibodies and antigens were determined using a Biacore T200 system (Cytiva). Antibodies were captured on a Protein A sensor chip (Cytiva) until the response units (RU) reached 400–600. Recombinant RBD proteins at serial concentrations were injected over the antibody‐coated surface at a flow rate of 30 µl/min in HBS‐EP+ buffer (10 mm HEPES, 150 mM NaCl, 3 mM EDTA, 0.05% surfactant P20; Cytiva). Association and dissociation phases were monitored for 450 s (RBD), respectively. The surface was regenerated with glycine‐HCl (pH 1.5) between cycles when necessary. Data were analyzed using the Biacore Evaluation Software with a 1:1 Langmuir binding model to calculate the kinetic rate constants (ka, kd) and the equilibrium dissociation constant.

## Author Contributions

H.S. and Y.L. conceptualized and designed the study. H.S. and H.L. designed and conducted the immunological experiments. H.S. and H.L. visualized the data. H.S. and Y.L. analyzed the data and prepared the manuscript. H.S. performed and analyzed the BCR sequencing results. H.L. expressed, purified, and validated the monoclonal antibodies. X.Z. sorted antigen‐specific B cells by flow cytometry. Z.Z. and D.R. constructed the HIS mice. D.R and S.D provided HSC for HIS mouse construction.Y.L. secured funding and supervised the study.

## Conflicts of Interest

Y. L. is the inventor of patents covering the development of humanized mouse models and therapeutic targeting of infection, cancer, autoimmune disorders. Among these, one patent application on human antibody development with HIS mice is relevant to this study.

## Supporting information




**Supporting File**: advs76276‐sup‐0001‐SuppMat.docx.

## Data Availability

The data that support the findings of this study are available from the corresponding author upon reasonable request.
