# Supplementary material for: Optimization of B Cell Responses in Human Immune System Mice Through Organoid Based Screening
Source: Adv Sci (Weinh). 2026 Jun 26:e24189. Online ahead of print. doi: 10.1002/advs.202524189 (PMC13337092; doi:10.1002/advs.202524189)
Supplement: Supplementary file 1 — Supporting File: advs76276‐sup‐0001‐SuppMat.docx. [file ADVS-9999-e24189-s001.docx]

Supporting Information for

# Optimization of B cell responses in human immune system mice through organoid based screening

Haiqiao Sun^1, #^, He Li^1, #^ Xu Zhu^1^, Zijian Zhang^1^, Deshan Ren^1^, Shuai Ding^2^ and Yan Li^1, 3,4,5*^

*Correspondence: yanli@nju.edu.cn (Y.L.)

**This PDF file includes:**

Figure S1 to S9

**
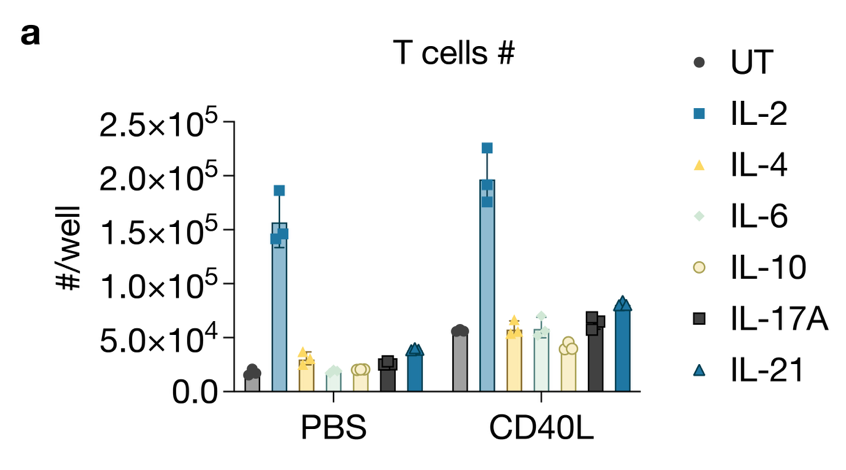
**

## Figure S1. IL-2 promotes extensive T cell expansion in HIS mouse spleen organoids.

a) T cells (T#) per well in HIS mouse spleen organoid cultures following treatment with various cytokines. The treatments include IL-2, IL-4, IL-6, IL-10, IL-17A, and IL-21, as well as PBS (UT) and CD40L. The data demonstrate that IL-2, both alone and in combination with CD40L, leads to a substantial expansion of T cells in the HIS mouse spleen organoid cultures.

**
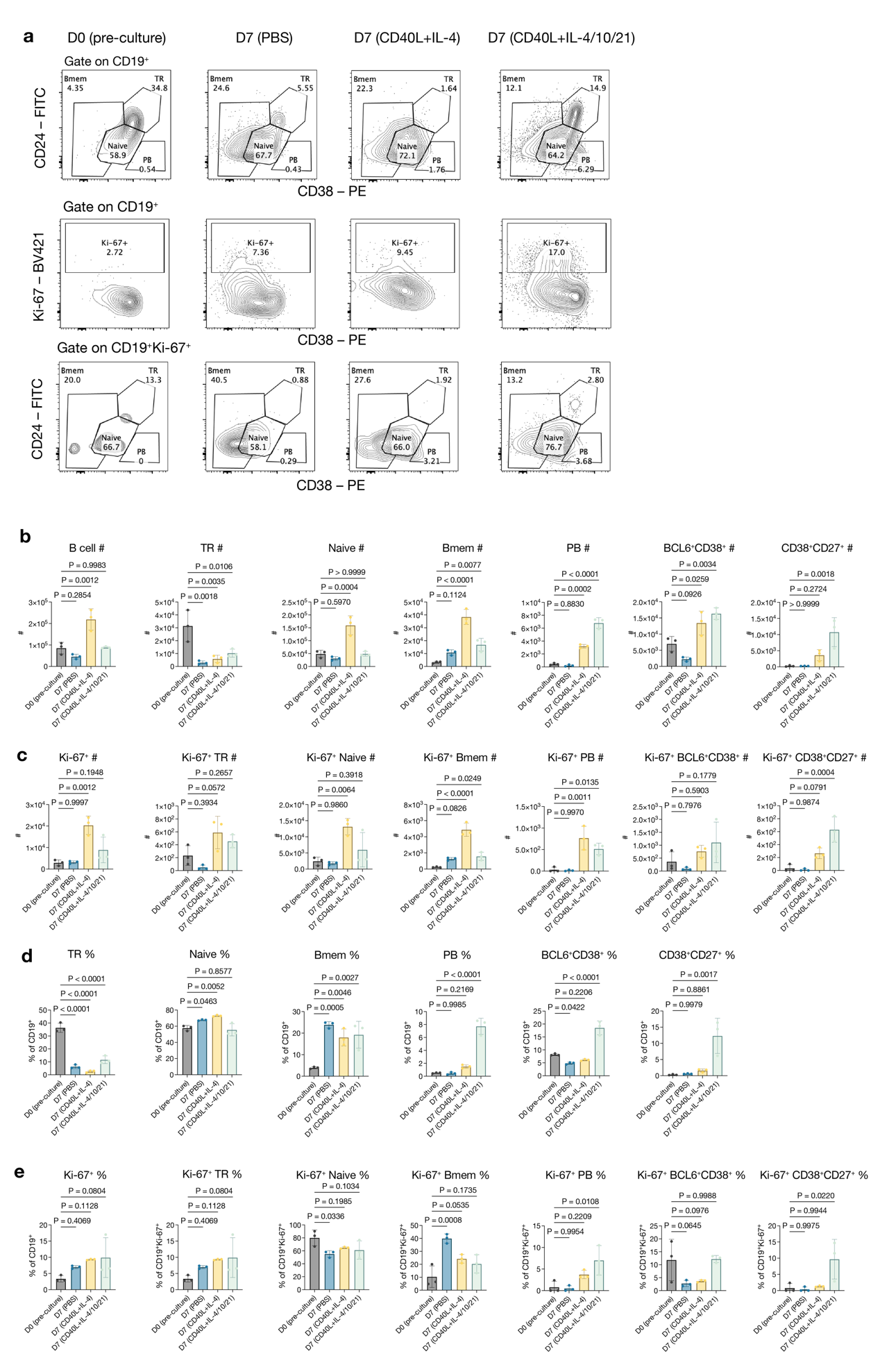
**

## Figure S2. Active proliferation and differentiation of B cells in HIS mouse SP organoids.

a) Representative flow cytometry plots of HIS mouse SP organoids at D0 (pre-culture) and at D7 under three conditions: PBS, CD40L+IL-4, and CD40L+IL-4/10/21. Top row: B cell subsets (TR, Naive, Bmem, PB) gated on CD19^+^ cells based on CD24 and CD38 expression. Middle row: Ki-67^+^ cells gated on CD19^+^. Bottom row: B cell subset distribution within the CD19^+^Ki-67^+^ proliferating population.

b) Absolute cell numbers of total B cells (CD19^+^) and B cell subsets (TR, Naive, Bmem, PB), as well as BCL6^+^CD38^+^ and CD38^+^CD27^+^ populations at D0 and D7 across treatment groups.

c) Absolute cell numbers of Ki-67+ cells within total B cells and within each subset (TR, Naive, Bmem, PB, BCL6^+^CD38^+^, CD38^+^CD27^+^) at D0 and D7 across treatment groups.

d) Proportions of B cell subsets ((TR, Naive, Bmem, PB, BCL6^+^CD38^+^, CD38^+^CD27^+^) within CD19^+^ B cells at D0 and D7 across treatment groups.

e) Proportions of Ki-67^+^ cells within total CD19^+^ B cells and within each subset at D0 and D7 across treatment groups. Statistical significance was calculated by one-way ANOVA. Data are presented as mean ± s.d.


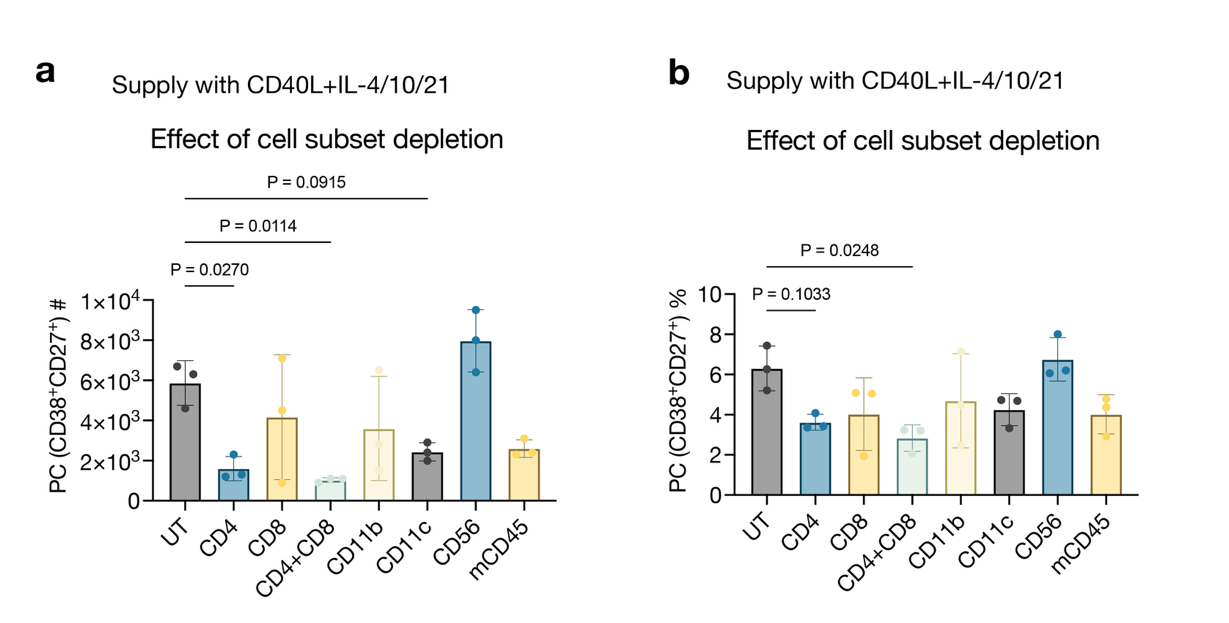


## Figure S3. Contribution of immune cell subsets to B cell survival and differentiation in HIS mouse SP organoids.

a) Quantification of PC numbers (CD38^+^CD27^+^) in SP organoids after depletion of the indicated cell subsets, cultured with CD40L+IL-4/10/21 for seven days.

b) Quantification of PC proportion (CD38^+^CD27^+^ as percentage of CD19^+^ cells) under the same conditions as (b). Data are presented as mean ± s.d. Statistical significance was calculated by one-way ANOVA. P < 0.05 was shown.


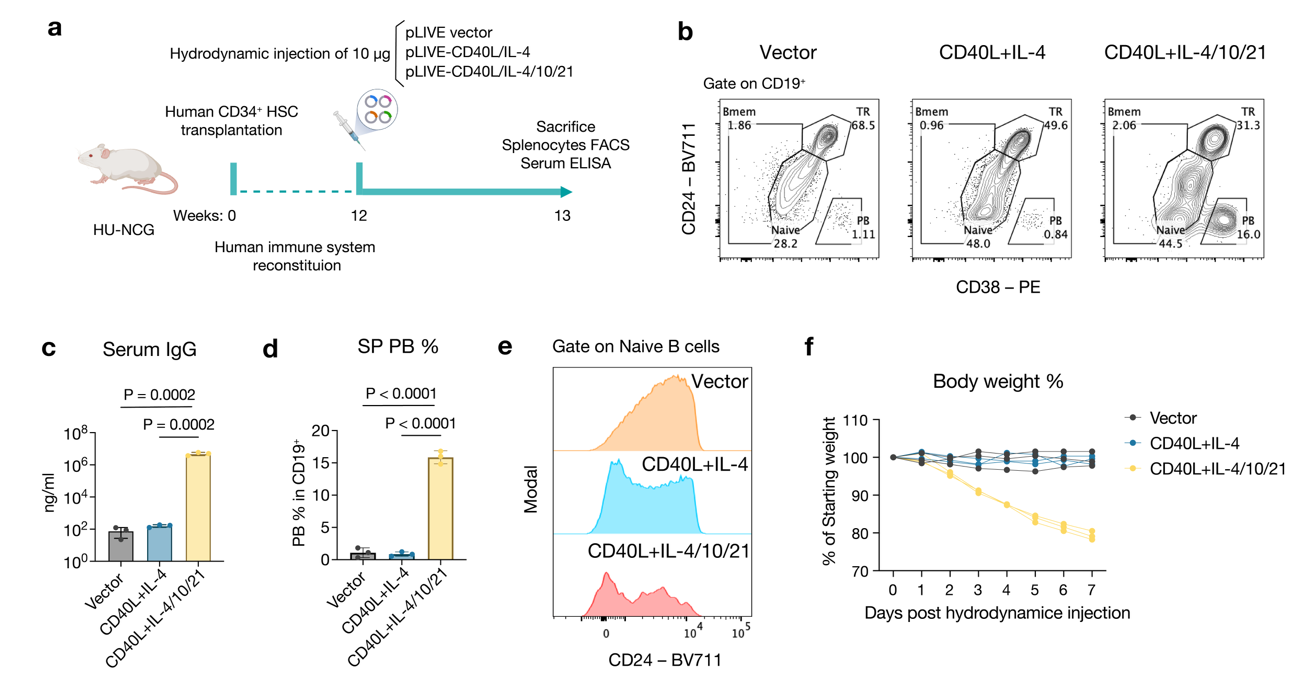


## Figure S4. Hydrodynamic injection of plasmids encoding second signals induces non-specific B cell differentiation and body weight in HIS mice.

a) Schematic of the experimental design for hydrodynamic injection of plasmids encoding second signal cytokines (IL-4, CD40L, or IL-4/10/21/CD40L) into HU-NCG mice. Plasmids (10 µg for each cytokine or 40 µg for the empty vector) were injected via hydrodynamic injection to express the cytokines and induce immune cell reconstitution and maturation (*n* = 3).

b) Representative flow cytometry analysis showing B cell subsets (Naive, TR, Bmem, and PB) in the SP of HIS mice 7 days after hydrodynamic injection.

c) Serum IgG levels measured by ELISA, showing significant increases in the IL-4/10/21/CD40L group.

d) Quantification of PB cells as a percentage of CD19^+^ B cells in the SP. The IL-4/10/21/CD40L group exhibited a significant increase in PB differentiation.

e) Flow cytometry analysis of Naive B cells in HIS mouse SP, showing CD24 downregulation in the IL-4/CD40L and IL-4/10/21/CD40L treatment groups.

f) Body weight changes in HIS mice post-hydrodynamic injection. Mice injected with IL-4/10/21/CD40L experienced a significant body weight reduction, with approximately 20% weight loss by day 7. Statistical significance was calculated by one-way ANOVA with Dunnett correction. Data are presented as mean ± s.d.


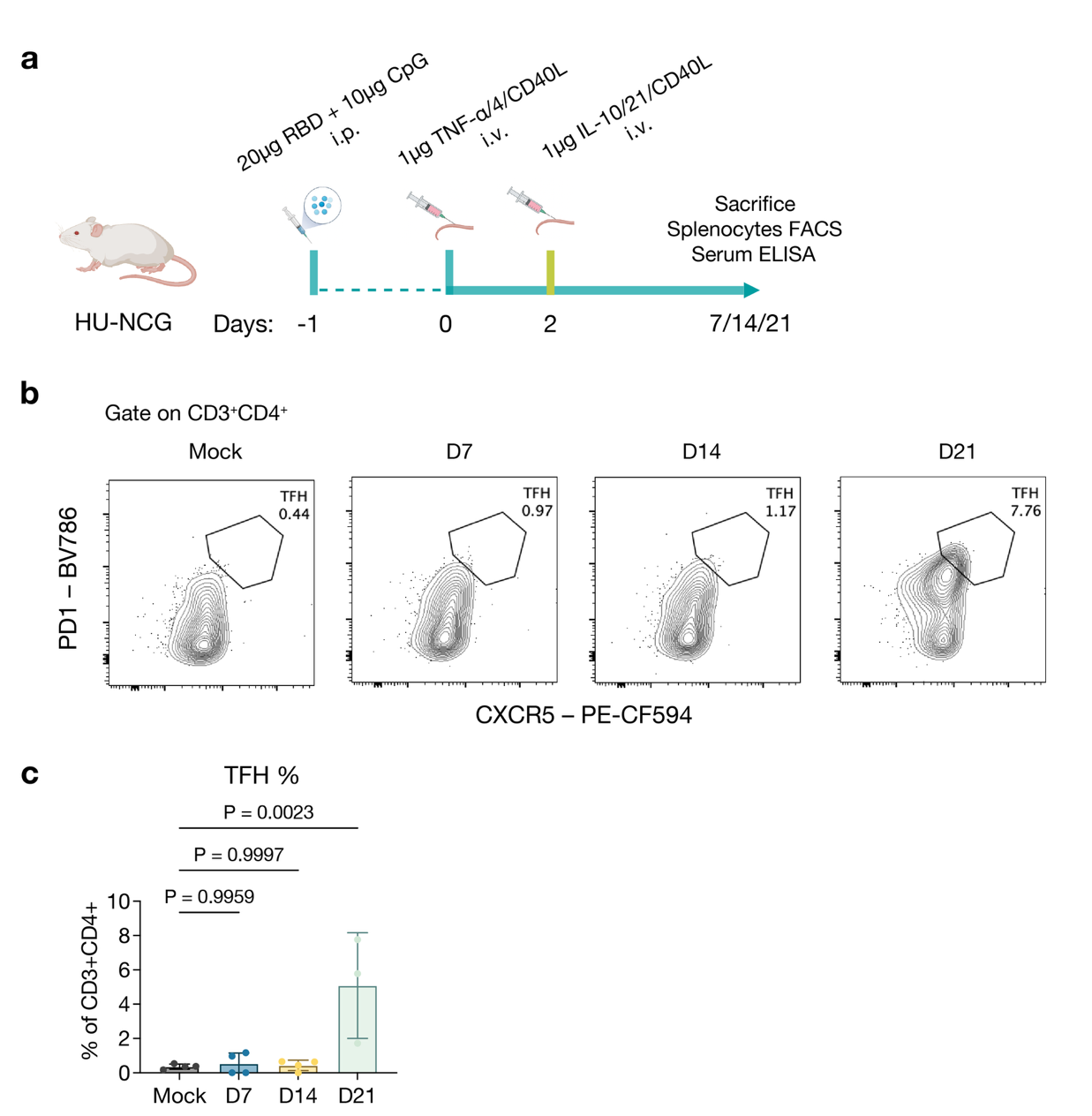


## Figure S5. Tfh cell analysis in SP of HIS mice following the optimized immunization protocol.

a) Schematic of the experimental design. HU-NCG mice were immunized with 20 µg of RBD protein and 10 µg of CpG adjuvant via i.p. injection at D-1. At D0, an expansion signal consisting of 1 µg each of TNF-α, IL-4, and CD40L was administered via i.v. injection. At D2, a differentiation signal consisting of 1 µg each of IL-10, IL-21, and CD40L was administered via i.v. injection. Mice were sacrificed at D7, D14, or D21 for SP FACS analysis and serum ELISA (*n* = 3-4).

b) Representative FACS plots showing Tfh cells (CXCR5^+^PD1^+^) gated on CD3^+^CD4^+^ T cells in SP at the indicated time points (Mock, D7, D14, D21). Numbers indicate the percentage of Tfh cells within the CD3^+^CD4^+^ gate.

b) Quantification of Tfh cell frequency as a percentage of CD3^+^CD4^+^ T cells at the indicated time points. Data are presented as mean ± s.d. Statistical significance was calculated by one-way ANOVA.


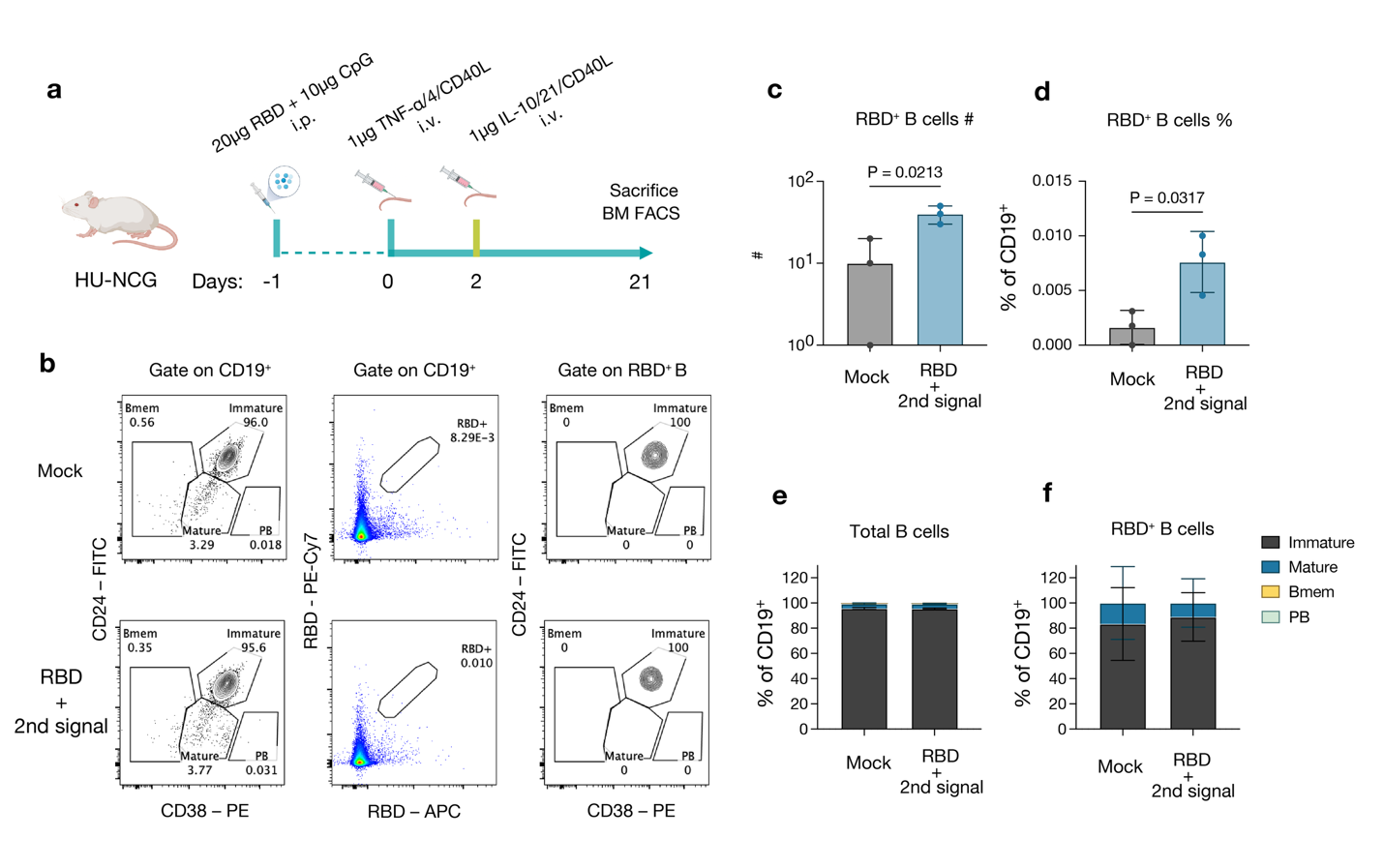


## Figure S6. Analysis of bone marrow B cells in HIS mice following immunization with second signal supplementation.

a) Schematic of the experimental design. HIS mice were immunized with 20 µg RBD and 10 µg CpG via i.p. at D-1, followed by expansion signal (1 µg each of TNF-α, IL-4, and CD40L, i.v.) at D0 and differentiation signal (1 µg each of IL-10, IL-21, and CD40L, i.v.) at D2. BM cells were harvested at D21 for FACS analysis (n = 3).

b) Representative flow cytometry plots showing total B cell subsets (left, gated on CD19^+^), RBD-specific B cell frequency (middle, gated on CD19^+^), and RBD-specific B cell subsets (right, gated on RBD^+^ B cells) in BM. B cell subsets were defined by CD24 and CD38 expression as immature (CD24^++^CD38^++^), mature (CD24^+^CD38^+^), Bmem (CD38^-^), and PB (CD24^-^CD38^+++^).

c) Quantification of total RBD-specific B cell numbers in BM.

d) Quantification of RBD-specific B cell frequency (as percentage of CD19^+^ cells) in BM.

e) Stacked bar graph showing the proportions of B cell subsets (Immature, Mature, Bmem, and PB) within total CD19^+^ B cells in BM.

f) Stacked bar graph showing the proportions of B cell subsets within RBD-specific B cells in BM. Data are presented as mean ± s.d. Statistical significance was calculated by unpaired t-test.


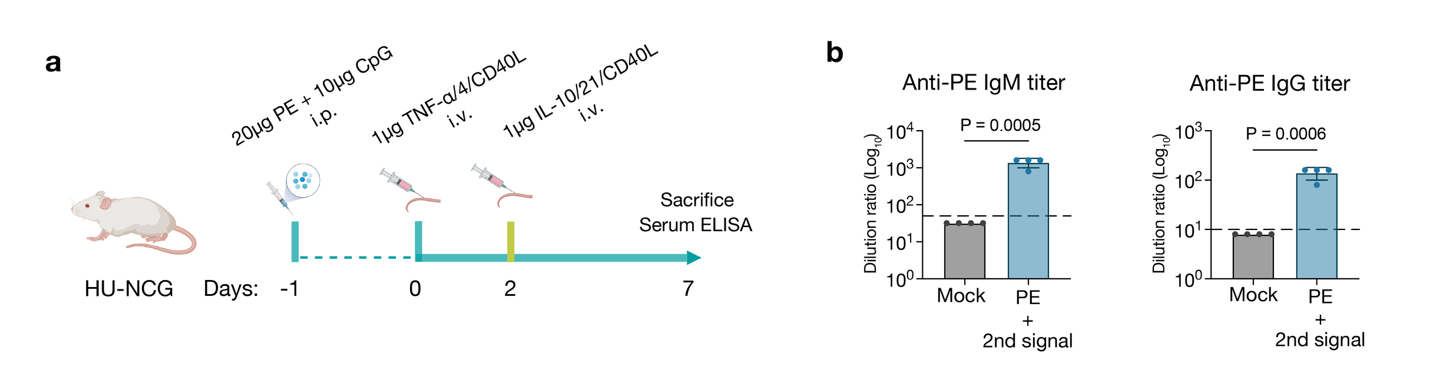


## Figure S7. Second signal supplementation promotes PE-specific B cell responses in HIS mice.

a) Schematic of the experimental design. HIS mice were immunized with PE protein and CpG adjuvant via i.p. injection, followed by second signal delivery via i.v. injection as described in Figure 7a. Mice were sacrificed at 7 dpi for SP FACS analysis and serum ELISA.

b) Quantification of PE-specific B cell frequency (as percentage of CD19^+^ cells) in SP at 7 dpi.

c) Anti-PE IgM titers in serum measured by ELISA. The dashed line indicates the limit of detection (LOD). Undetectable values were set to LOD minus 0.2 log units. Data are presented as mean ± s.d. Statistical significance was calculated by unpaired t-test.


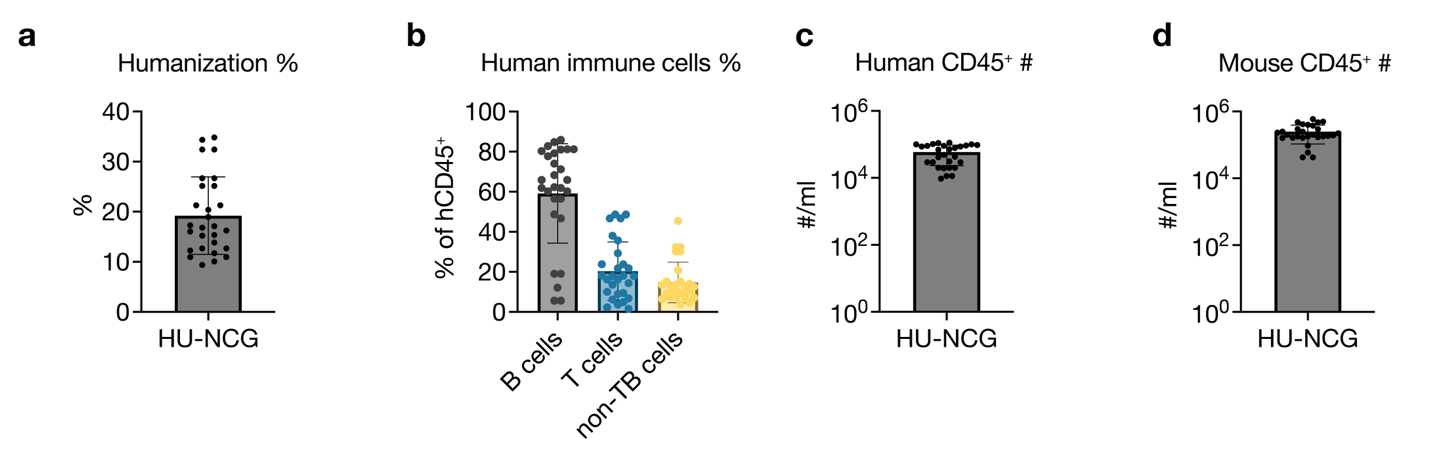


## Figure S8. Quality control of humanization efficiency in peripheral blood of HIS mice.

a) The humanization percentage in peripheral blood HU-NCG mice was measured by flowcytometry. The humanization percentage was calculated as hCD45^+^/ (hCD45^+^ + mCD45^+^) * 100%.

b) The proportion of human immune cells, including B cells, T cells, and non-TB cells, among hCD45^+^ cells was also assessed.

c) The total number of human CD45^+^ cells per milliliter of peripheral blood is presented.

d) The number of mice CD45^+^ cells per milliliter of peripheral blood is also shown for comparison.


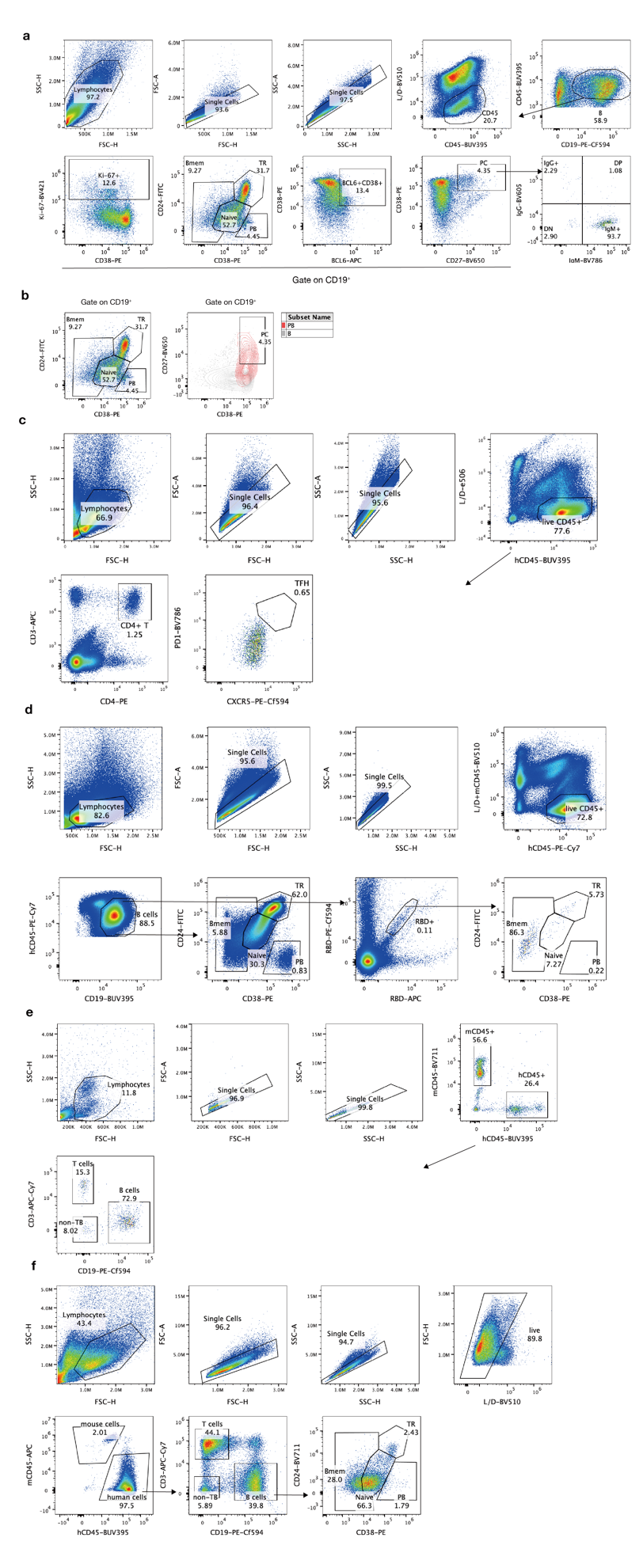


## Figure S9. Flow cytometry gating strategy.

a) Gating strategy for intracellular staining of B cell subsets in HIS mouse spleens.

b) Mapping plot of the CD38⁺CD24^-^ subset within the CD38/CD27 gating strategy.

c) Gating strategy for Tfh cells in HIS mouse spleens.

d) Gating strategy for RBD-specific B cells in HIS mouse spleens. After lymphocyte gating, CD19^+^ B cells are identified, followed by gating on CD24 and CD38 for identifying different B cell subsets (Naive, TR, Bmem, and PB). RBD-specific B cells are then identified by RBD binding (RBD^+^). Gating was performed in a left-to-right, top-down sequence.

e) Gating for humanization percentage of HIS mice in peripheral blood.

f) The differentiation of human B cells into TR, Naive, Bmem, and PB subsets.
